# Global migration and the changing distribution of sickle haemoglobin: a quantitative study of temporal trends between 1960 and 2000

**DOI:** 10.1016/S2214-109X(13)70150-5

**Published:** 2014-02

**Authors:** Frédéric B Piel, Andrew J Tatem, Zhuojie Huang, Sunetra Gupta, Thomas N Williams, David J Weatherall

**Affiliations:** a Evolutionary Ecology of Infectious Disease Group, Tinbergen Building, Department of Zoology, University of Oxford, Oxford, UK; b Global Sickle Cell Disease Network, Toronto, ON, Canada; c Department of Geography and Environment, University of Southampton, Southampton, UK; d Fogarty International Center, National Institutes of Health, Bethesda, MD, USA; e Center for Infectious Disease Dynamics and Department of Biology, Pennsylvania State University, PA, USA; f Kenya Medical Research Institute–Wellcome Trust Programme, Centre for Geographic Medicine Research-Coast, Kilifi District Hospital, Kilifi, Kenya; g Department of Medicine, Imperial College, St Mary's Hospital, London, UK; h Weatherall Institute of Molecular Medicine, University of Oxford, Oxford, UK

## Abstract

**Background:**

Changes in the geographical distribution of genetic disorders are often thought to happen slowly, especially when compared with infectious diseases. Whereas mutations, genetic drift, and natural selection take place over many generations, epidemics can spread through large populations within a few days or weeks. Nevertheless, population movements can interfere with these processes, and few studies have been done of their effect on genetic disorders. We aimed to investigate the effect of global migration on the distribution of the sickle-cell gene—the most common and clinically significant haemoglobin structural variant.

**Methods:**

For each country, we extracted data from the World Bank's Global Bilateral Migration Database about international human migrations between 1960 and 2000. We combined this information with evidence-based estimates of national HbS allele frequencies, generated within a Bayesian geostatistical framework, to analyse temporal changes in the net numbers of migrants, and classified countries with an index summarising these temporal trends.

**Findings:**

The number of international migrants increased from 92·6 million in 1960, to 165·2 million in 2000. The estimated global number of migrants with HbS increased from about 1·6 million in 1960, to 3·6 million in 2000. This increase was largely due to an increase in the number of migrants from countries with HbS allele frequencies higher than 10%, from 3·1 million in 1960, to 14·2 million in 2000. Additionally, the mean number of countries of origin for each destination country increased from 70 (SE 46) in 1960, to 98 (48) in 2000, showing an increasing diversity in the network of international migrations between countries. Our index of change map shows a patchy distribution of the magnitude of temporal changes, with the highest positive and negative values scattered across all continents.

**Interpretation:**

Global human population movements have had a substantial effect on the distribution of the HbS gene. Population movements can create a long-term burden on health-care systems. Our findings, which emphasise countries in which migration fluxes are changing the most, should increase awareness about the global burden of haemoglobinopathies and encourage policy makers to implement specific public health interventions, such as screening programmes and genetic counselling.

**Funding:**

Wellcome Trust, European Research Council, Bill & Melinda Gates Foundation, National Institute of Allergy and Infectious Diseases–National Institutes of Health, the Research and Policy for Infectious Disease Dynamics program, Fogarty International Center.

## Introduction

The mobility of human beings worldwide has reached an unprecedented level.^1^ With changes in the modes and speed of international transportation, constraints previously imposed by natural barriers and long distances have greatly reduced. This increasing globalisation means that populations previously isolated from one another are now linked by regular fluxes of travellers and migrants.^2^ This process has had a substantial effect on public health.^3^, ^4^, ^5^, ^6^ With increasing mobility over long distances, the risks of local disease outbreaks spreading globally have become much higher than previously, as shown by recent influenza pandemics and spread of vector-borne diseases.^7^, ^8^, ^9^ International migrations can also have a long-term effect on public health through the introduction of deleterious genes into populations in which they were previously absent. Because genes are inherited over generations, their burden can seem to present an indirect threat to public health, and few studies have focused on changes in the distribution of genetic disorders.^10^, ^11^, ^12^ However, as shown for infectious diseases, prevention measures based on early diagnosis can reduce the long-term burden of genetic disorders,^13^ and regular assessment of the associated risks, on the basis of existing epidemiological knowledge,^14^ is essential.

After decades of neglect, sickle haemoglobin (HbS) is emerging as a major public health concern.^15^ HbS is a structural variant of normal adult haemoglobin (HbA) that is inherited as an autosomal recessive mendelian trait. Whereas individuals heterozygous for sickle haemoglobin (HbAS) are generally asymptomatic, most homozygous children (HbSS) die before the age of 5 years in low-income countries, and most surviving adults suffer from lifelong acute and chronic complications in high-income countries.^16^, ^17^ As originally suggested by the so-called malaria hypothesis, the geographical distribution of HbS was historically driven by the selective advantage conferred by this gene in protecting against *Plasmodium falciparum* malaria infection in heterozygous individuals.^18^, ^19^ HbS was therefore identified in malarious regions across sub-Saharan Africa; parts of the Mediterranean, including Greece and southern Turkey; various oases on the eastern and western coast of the Arabian Peninsula; and in Indian tribes in the southern part of Chhattisgarh and Karnataka.^20^, ^21^, ^22^ HbS was absent in Indigenous Americans, northern Europeans, and Oceanian populations.^20^, ^21^, ^22^ Distribution of the HbS gene expanded substantially after forced migration of African people to the Americas through the African slave trade.^23^ As such, HbS became relatively prevalent in African American populations, particularly in those in the USA,^24^ Brazil,^25^ and the Caribbean.^26^

The health burden of haemoglobinopathies, including sickle haemoglobin, is expected to increase in the coming decades.^27^, ^28^ The number of individuals needing diagnosis and management for these disorders is increasing in both low-income and high-income countries. In low-income countries, substantial improvements in nutrition, hygiene, and access to health care and drugs have led to important reductions in childhood mortality, which increase the chances of survival of children with sickle-cell anaemia, who would have previously died undiagnosed.^29^ In high-income countries, immigration from countries with a high HbS prevalence contribute to the spread of this gene into new populations, within which screening programmes become increasingly necessary.^24^, ^30^ In both cases, clinicians are becoming increasingly likely to encounter HbS patients or carriers, and need to be able to provide adequate health care and counselling. Awareness of the disease, combined with efficient prevention programmes, based on appropriate diagnosis and management, can help greatly in the reduction of long-term severe complications.^17^

In this study, we aimed to investigate the effect of recent international population movements on the distribution of the sickle-cell gene. We assess the main migration trends and define countries in which the implementation of appropriate public health policies might be more urgently needed on the basis of the origins of migrants. Although we focus on HbS, various other common haemoglobin variants, including HbC, HbE, HbD, and a large range of α-thalassaemia and β-thalassaemia mutations, are also spreading globally because of increasing population movements, and the same principles as for HbS apply.

## Methods

### Database and data extraction

We extracted data from the World Bank's Global Bilateral Migration Database.^31^ This database includes decennial matrices of migrant stocks for the period 1960–2000 for 232 countries. It provides a comprehensive picture of human migrations worldwide on the basis of censuses and population registry records, with migrant origins defined by their country of birth. Various techniques were used to estimate missing data to provide a single and complete matrix of international bilateral migrant stocks.^31^ No measures of uncertainty associated with this dataset are available. We then combined this information with recent evidence-based estimates of national HbS allele frequencies (posterior median), with uncertainty measures (IQR), generated within a Bayesian geostatistical framework.^32^

### Statistical analysis

In view of the usual clinical severity of the disease and shortened life expectancy of HbSS individuals, we assumed that migrants would have either normal haemoglobin (HbAA) or would be carriers of the sickle-cell trait (HbAS). We used the Hardy-Weinberg equilibrium equation^33^, ^34^ to estimate the level of HbS gene importation into each destination country, and calculated the estimated number of net HbAS individuals who had moved in and out of each country in 1960, 1970, 1980, 1990, and 2000.

We calculated the net number of migrants and estimated the net number of migrants with HbS as the difference between the estimated number of immigrants with HbS and the estimated number of emigrants with HbS (appendix). To enable comparison of trends in the estimated net number of migrants with HbS with those in the net number of overall migrants globally, or for a specific country, we used the number of migrants at the start of the study period (ie, 1960) as a reference value, and converted values for subsequent years into percentage differences. We generated temporal plots of these relative changes in the net number of migrants and estimated net number of migrants with HbS for each country (appendix). We then classified countries into seven classes on the basis of a combination of two factors. The first was the HbS index, which is based on the slope of a linear regression fitted to the net number of migrants with HbS. A positive slope shows an increasing burden, whereas a negative slope suggests a decreasing burden. If the slope was more than 2, the code given was +1. If the slope was between −2 and 2, the code given was 0, and if the slope was −2 or less, the code given was −1. The second factor was the DIV index, which categorises the divergence between trends in the number of net migrants and the estimated number of net migrants with HbS, as assessed by the absolute value of the ratio (termed *R*) or difference (termed *D*) between the slope of the two trend lines, termed HbS slope and MIG slope, respectively, dependent on the sign of the two slopes (appendix). An increasing distance between the two trend lines shows a growing change in the relative burden of net migrants with HbS compared with the net flux of overall migrants. If the decrease in the net number of estimated migrants with HbS is lower than that in the number of net migrants, or if the increase is higher, the burden will increase; conversely, if the decrease in the net number of migrants with HbS is greater than that in the number of net migrants, or if the increase is lower, the burden will decrease. The appendix summarises the rules used to define DIV index. We defined cutoff values for each combination of slope signs with quantiles of the normalised distribution of *R* or *D,* whichever was appropriate. We used a maximum of five classes, from −2 to +2, for the DIV index. Finally, we calculated a summary index—the index of change—as the sum of the HbS and DIV indices. As such, the values of the index of change range between −3 and 3. The appendix shows values of the HbS and MIG slopes, HbS index, DIV index, and index of change for all countries and gives further details about the calculation of the index of change, with reference to some specific working examples.

Maps of the estimated net number of migrants with HbS at the country level and the map of our overall index of change were created in ArcMap (version 10.1). Maps of fluxes to and from selected countries were generated with the tailored code written in R 2.15.2^35^ (appendix) and implemented in ArcMap (version 10.1).

### Role of the funding source

The sponsors of the study had no role in study design, data collection, data analysis, data interpretation, or writing of the report. The corresponding author had full access to all the data in the study and had final responsibility for the decision to submit for publication.

## Results

**Figure 1:**
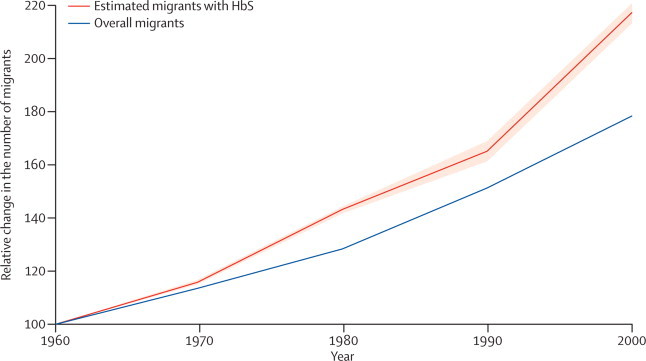
Global trends in the number of international migrants and estimated migrants with HbS compared with the 1960s level We calculated the solid red line on the basis of median HbS frequency; the light red area represents the uncertainty on the basis of the 25% and 75% quantiles. HbS=sickle-cell haemoglobin.

**Figure 2:**
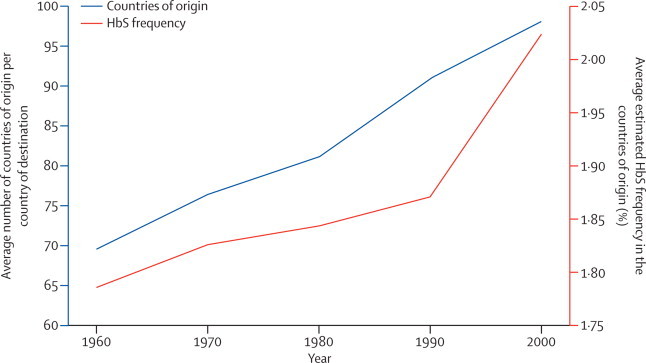
Global trends in the average number of countries of origin per country of destination and the average estimated HbS frequency in the countries of origin per country of destination between 1960 and 2000 HbS=sickle-cell haemoglobin.

Globally, the number of international migrants increased from 92·6 million in 1960, to 165.2 million in 2000.^1^ In that same period, the estimated number of migrants with HbS increased faster than did the overall number of migrants, from about 1·6 million in 1960, to 3·6 million in 2000 (figure 1). This finding is due to an increase in the estimated number of migrants from countries with HbS allele frequencies higher than 10%, from 3 083 457 in 1960, to 14 178 197 in 2000. Alongside this increase, the mean number of countries of origin for each destination country increased from 70 (SE 46) in 1960, to 98 (48) in 2000, showing an increasing diversity in the network of international migrations between countries. This trend was also accompanied by an increase in the mean overall frequency of HbS in the countries of origin, from 1·79% (SE 1·30) in 1960, to 2·02% (1·12) in 2000 (figure 2).

**Figure 3:**
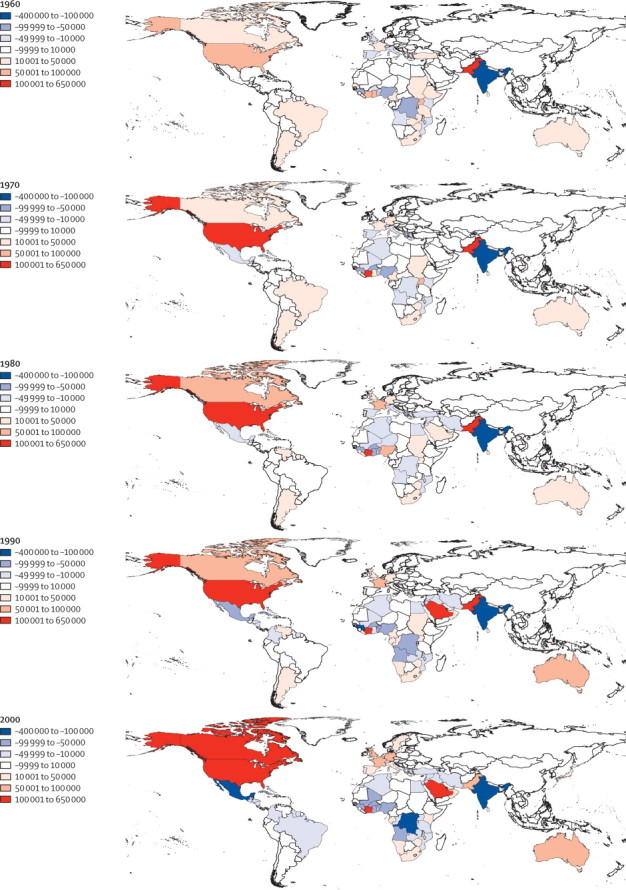
Estimated net migrations of individuals with HbS at the country level in 1960, 1970, 1980, 1990, and 2000 Countries in red correspond to those in which the estimated number of immigrants with HbS is higher than the estimated number of emigrants with HbS (sink countries). Countries in blue correspond to those in which the estimated number of immigrants with HbS is lower than the estimated number of emigrants with HbS (source countries). To aid comparisons, the same colour classes were used for all maps. HbS=sickle-cell haemoglobin.

The maps in figure 3 emphasise the changes in the effect of international migrations on the distribution of HbS globally between 1960 and 2000. The estimated number of migrants with HbS moving to North America, western Europe, or Australia increased (so-called sinks of HbS), whereas trends in African countries, India, and the Middle East were towards negative net fluxes of migrants with HbS (so-called sources). Because of the size of its population, India remained one of the main source countries throughout the study period (figure 3).

**Figure 4:**
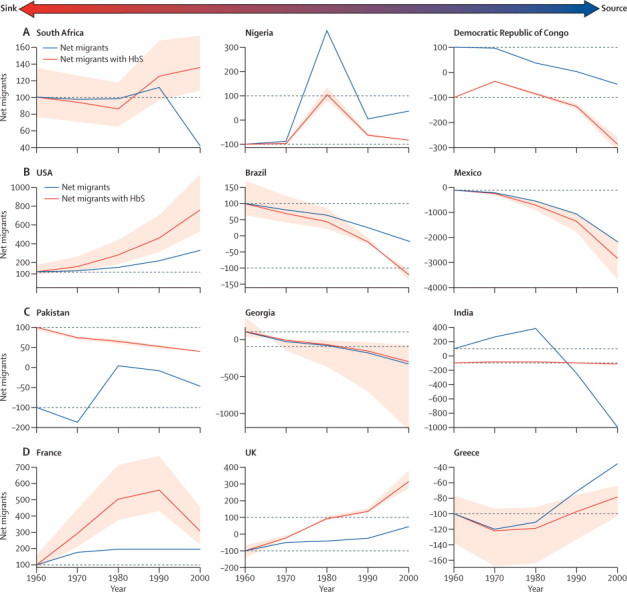
Net numbers of migrants and estimated net number of migrants with HbS (based on the posterior median) relative to the 1960 level for selected countries in Africa (A), the Americas (B), Asia (C), and Europe (D), between 1960 and 2000 Countries on the left-hand side tend to be sinks of migrants with HbS (ie, positive values along the y-axis); countries on the right-hand side tend to be sources of migrants with HbS (ie, negative values along the y-axis). Uncertainties (based on the IQR) associated with the trends in migrants with HbS are shown in light red. The appendix shows plots for all countries. HbS=sickle-cell haemoglobin.

By analysis of the net number of overall migrants and the net number of migrants with HbS, relative to their respective levels in 1960 for each country, we could examine specific trends at the national level (appendix). Figure 4 shows subregional variations for a subset of source and sink countries within each region. In Africa, HbS was originally common in the Democratic Republic of Congo (source), but was relatively rare in South African populations (sink). Changes in South Africa seem to have happened mostly in the 1990s (figure 4). In the Americas, the patterns between Mexico and the USA, in which the HbS gene was first introduced via slave trade routes, are contrasting. In Mexico, the number of net migrants and estimated number of net migrants with HbS are both decreasing, but the number of net migrants is decreasing more slowly than the estimated number of net migrants with HbS, suggesting a decreasing burden in relation to migrations (figure 4). In the USA, both trend lines are increasing and the number of net migrants with HbS is increasing faster than the overall net number of migrants, suggesting an increasing burden in relation to migrations (figure 4). Across Asia, although estimated fluxes of migrants with HbS mostly decreased during the study period, net relative fluxes were positive in Pakistan (sink), but negative in India (source), which indicates the regions of high prevalence in parts of the Indian subcontinent. In Europe, western European countries were overall sink countries (eg, France and UK), whereas Mediterranean countries where HbS is endemic were usually source countries (eg, Greece; figure 4). Patterns recorded in other countries varied (appendix).

**Figure 5:**
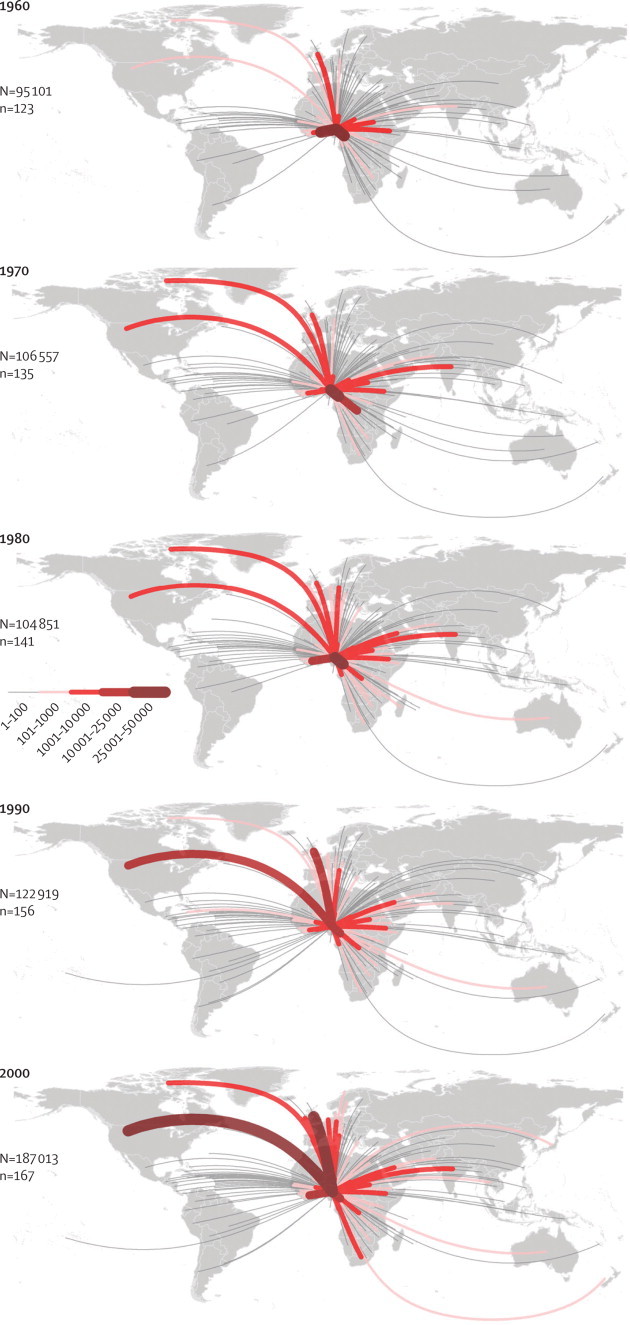
Estimated migration fluxes of individuals with HbS from Nigeria between 1960 and 2000 Thickness of the lines is proportional to the estimated number of HbS migrants to a given country. N=estimated number of net migrants with HbS. n=the number of countries to which individuals migrated. HbS=sickle-cell haemoglobin.

**Figure 6:**
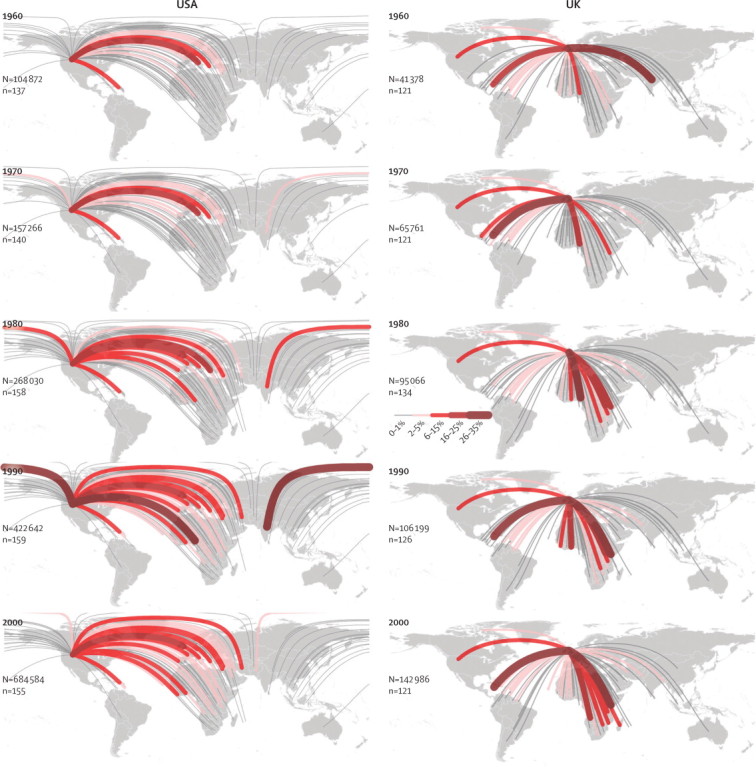
Estimated migration fluxes of individuals with HbS to the USA and the UK from 1960 to 2000 The thickness of the lines is proportional to the proportion of HbS migrants from a specific country amongst the total number of HbS immigrants. N=the estimated number of net migrants with HbS. n=the number of countries from which individuals migrated. HbS=sickle-cell haemoglobin.

Our index of change provides a summary of the main trends globally (appendix). This map shows a patchy distribution of the magnitude of temporal changes, with the highest positive (eg, Ecuador, Botswana, Portugal, and Thailand) and negative values (eg, Panama, Albania, Madagascar, and Iran) scattered in all continents. Estimated outgoing fluxes of migrants with HbS from Nigeria—the country with the highest burden of sickle-cell disease—increased quantitatively between 1960 and 2000, and there was diversification in their destinations (figure 5). Similar trends were shown in assessment of estimated incoming fluxes of migrants with HbS to the USA and the UK—two sink countries, both of which have implemented screening programmes for sickle-cell disorders (figure 6).

## Discussion

Our findings emphasise the spread of the HbS gene through recent human migrations and the global importance of this emerging public health problem. Although the magnitude of the public health burden associated with HbS in a given country is mainly driven by the frequency of this gene in its native and assimilated populations,^32^ clinicians worldwide should be aware that the possibility of encountering a patient with symptoms associated with sickling disorders is increasing.^36^, ^37^, ^38^, ^39^, ^40^, ^41^, ^42^ Our summary map (appendix) draws attention to countries in which these changes are happening, and therefore, to those where a focus on awareness and prevention, through the introduction or enhancement of screening programmes, should become a priority.^13^ The USA and UK have both implemented universal newborn screening programmes,^30^, ^43^, ^44^ but screening of immigrants is less systematic. Various European countries, including the Netherlands,^45^ France,^46^ Greece,^47^ and Cyprus,^48^ have implemented registries for haemoglobinopathies, but integration of immigrant patients to those specific health-care services is still often difficult.^10^, ^49^ In Africa, large-scale screening programmes are still uncommon.

In the absence of a curative treatment for sickle-cell disorders,^50^ and in view of the huge lifetime costs associated with the treatment of patients with these disorders,^51^ education, planning policies, and prevention programmes are essential strategies to reduce the subsequent long-term economic and health burden.^27^, ^28^, ^29^ Rather than suggesting strict immigration policies, our findings emphasise the need for an international approach to prevent such genetic health disorders globally. Although WHO recognised sickle-cell disease as a global public health problem in 2006,^15^ and a resolution on the prevention and management of birth defects, including sickle-cell disease and the thalassaemias, was adopted in 2010 at the 63rd World Health Assembly, little has changed in terms of international public health policies. Until such changes take place, collaborations between source and sink countries could be developed on the basis of political, cultural, religious, and linguistic similarities, and historical links between countries.^2^ For example, the links between the Democratic Republic of Congo and Belgium, or France and Algeria, are clearly shown in their migration patterns. Those links could be developed to educate and prevent sickle-cell disorders in source countries, which would be of direct benefit to the sink countries, and to lessen the effect of linguistic, cultural, and social barriers, which are usually associated with immigration in sink countries. For example, immigrants often tend to stay endogamous, and consanguineous marriages are common practice in various ethnic groups. Understanding of social behaviours is therefore crucial in the prevention of these disorders.^52^, ^53^, ^54^ The research community has already started developing such a collaborative approach, as shown by the Global Sickle Cell Disease Network, which aims to bring together clinicians and researchers from all over the world to build a dynamic community focusing on a global health problem, increase awareness of sickle-cell disorders, and improve the future of patients; however, similar official collaborations supported by ministries of health, international health agencies, and major funding bodies have so far been scarce.^55^

The trends described in this study are also noted for many other mutations of the globin genes, including HbC, HbE, HbD, and numerous α-thalassaemia and β-thalassaemia variants, which should also be carefully considered. Many haemoglobin and thalassaemia variants have already been identified and, thanks to advances in genetics, new variants are still regularly being discovered.^56^ Larger and more diverse fluxes of migrants could result in the emergence of pairs of variants, which were previously highly unlikely to be co-inherited because of the absence of overlap between their respective original geographical distributions, leading to an increasing complexity of genotypes and phenotypes potentially encountered by clinicians.^57^ Increased awareness about known variants and their severity is crucial in the prevention of the most severe combinations.

Our study has several limitations. First, we assumed that the probability to emigrate is equal for any individual within a specific country, and that this probability is independent of the ethnic origin or HbS genotype of a particular individual. This assumption is especially relevant in a country such as India in which the social structure is very complex and would certainly affect the opportunities to migrate. In the absence of data for these parameters for migrants, a stochastic model-based approach could be developed to account for different likelihoods to migrate. Such an approach would also enable the generation of precision measures associated with the trends shown in this analysis. Second, the accuracy of the international migration database used here is unknown. A complex succession of techniques is used to standardise the data and to fill in the gaps in space and time.^31^ Moreover, some official migration data are based on random surveys (eg, the international passenger survey in the UK) and illegal migrations are not included. Nevertheless, no associated uncertainty measures are available. The uncertainties we report are therefore only associated with the HbS frequency estimates. Studies of global migration have only been undertaken recently^1^, ^2^ and an increasing demand for reliable and comprehensive data might lead to the calculation of precision measures in future international migration databases.^58^, ^59^ Third, this study focuses on only sickle haemoglobin; information about other haemoglobinopathies or birth defects would be needed to better assess the effect of migrants on local public health services. Compound statuses like sickle-cell haemoglobin C disease or sickle-cell β thalassaemia also contribute to the burden of sickle haemoglobin. This limitation is partly due to our restricted knowledge of the contemporary distribution and prevalence of such compound statuses. Finally, whether the trends observed over the last half-century will continue in the coming years is unclear,^60^ and the implementation of specific health interventions should be carefully considered. Studies have been published about the cost-effectiveness of targeted versus universal screening programmes,^43^, ^61^ and this effectiveness will depend on the individual context of each country. Nevertheless, increased awareness about this disorder and its wide distribution can only benefit patients and carriers.

This study provides further evidence of the fact that public health problems represent global issues that need to be addressed collectively by low-income and high-income countries (panel).^4^, ^36^ The likelihood of finding diseases and genetic disorders that previously had a restricted distribution has increased in recent decades, and this trend is unlikely to change in the near future. Countries can adopt national measures, such as screening programmes, to reduce the local burden of such disorders, but in the long term, a concerted approach based on multinational collaborations and partnerships focusing on countries of high prevalence would probably be much more effective;^29^ this approach is particularly relevant for sickle-cell disorders.

Panel **Research in context**

**Systematic review**

The search strategy and selection criteria have been previously described.^32^ Although various studies have described the effects of globalisation and population movements from a social, cultural, economic, or political point of view, few have investigated the effect of recent population movements on the distribution of human deleterious genes. Such migrations can have a profound effect on the range and frequency of inherited disorders. Angastiniotis and colleagues studied the effect of migrations on health services for rare diseases across Europe.^10^ In the specialty of haemoglobinopathies, various studies have been done investigating the effect of migration on health-care services for patients with sickle-cell disease and thalassaemia in countries including the UK,^61^ the USA,^14^ Western Australia,^62^ Iran,^63^ and Italy.^36^ Nevertheless, the effect of recent international migrations on the global distribution of the sickle-cell gene^32^ has not been investigated in detail.

**Interpretation**

To our knowledge, this study is the first to investigate the effect of recent global migration on the distribution of a deleterious gene—the sickle-cell gene. The increase and diversification of migration fluxes and their effect on health-care systems should be seriously considered when national and international health policies are defined.

## Supplementary Material

Supplementary appendix

Supplementary audio Frédéric Piel discusses the effect of global migration on the distribution of the sickle-cell gene.
