# Supplementary material for: Global migration and the changing distribution of sickle haemoglobin: a quantitative study of temporal trends between 1960 and 2000
Source: Lancet Glob Health. 2014 Feb;2(2):e80–9. doi: 10.1016/S2214-109X(13)70150-5 (PMC3986033; doi:10.1016/S2214-109X(13)70150-5)
Supplement: Supplementary appendix [file mmc1.pdf]

THE LANCET Global Health

Supplementary appendix

This appendix formed part of the original submission and has been peer reviewed. We post it as supplied by the authors.

Supplement to: Piel FB, Tatem AJ, Huang Z, Gupta S, Williams TN, Weatherall DJ. Global migration and the changing distribution of sickle haemoglobin: a quantitative study of temporal trends between 1960 and 2000. *Lancet Glob Health* 2014; published online Jan 14. http://dx.doi.org/10.1016/S2214-109X(13)70150-5.

Supplementary Appendix

Supplement to: Piel, F.B., Tatem, A.J., Huang, Z., Gupta, S., Williams, T.N. and Weatherall, D.J. 2014 Global migration and the changing distribution of sickle haemoglobin: a quantitative study of temporal trends between 1960 and 2000. *Lancet Global Health*. doi: 10.1016/S2214-109X(13)70150-5.

Supplementary Materials

Supplementary Material 1 Calculation of our Index of Change.

Supplementary Material 2 R code used to generate the flow maps.

Supplementary Figures

Supplementary Figure 1 Flowchart summarising the approach used to calculate our Index of Change. Net numbers have been calculated as the difference between the number of immigrants and emigrants. Relative numbers reflect the migrant flows compared to their reference level in 1960 (100). MIG slope represents the slope of the linear regression fitted to the relative net number of migrants. HbS slope represents the slope of the linear regression fitted to the estimated relative net number of migrants with HbS. HbS Index represents a coded index of HbS slope in three classes. Ratio (R) is the ratio between MIG slope and HbS slope. DIV Index is the divergence index between the two trend lines fitted to the relative net numbers of migrants and migrants with HbS, respectively. DIV index values ranging from -2 to 2 were given based on the following cut-off values for *R*: 0.50, 0.60, 1.35 and 2.00. The Index of Change is the sum of HbS Index and DIV Index.

Supplementary Figure 2. Divergence index calculation rules. MIG slope (in blue) = slope of the trend in the number of overall net migrants between 1960 and 2000; HbS slope (in red) = slope of the trend in the estimated number of net migrants with HbS between 1960 and 2000; *R* = absolute ratio between HbS slope and MIG slope; *D* = absolute difference between HbS slope and MIG slope. n= number of countries falling within each of the quadrants. The values of the divergence index are shown in bold. See Supplementary Material A for further details.

Supplementary Figure 3 Relative net numbers of migrants (blue line) and estimated relative net number of migrants with HbS (red line) compared to the 1960 level for all countries. Estimates based on the posterior median of HbS predictions published in Piel *et al* 2013. The red shaded area reflects the uncertainty (i.e. interquartile range) in the HbS predictions.

Supplementary Figure 4 Map of our Index of Change. Classification of countries based on absolute trends in the net number of migrants with HbS during the study period (1960-2000) and relative trends compared to the overall net number of migrants. The index is based on the slope of the trend line for net HbS migrants (HbS Index) and the divergence between the trend lines for net HbS migrants and net overall migrants (DIV Index). A positive Index, shown in red, suggests that the estimated net flux of migrants with HbS has proportionally increased over the study period and that implementing specific interventions for the prevention of sickle cell disease should likely be considered. A negative Index, shown in blue, suggests that

the estimated net flux of migrants with HbS has proportionally decreased over the study period and that specific interventions for the prevention of sickle cell disease are unlikely to be a health priority.

Supplementary Tables

Supplementary Table 1 Slope coefficients for the overall net migrant (MIG) and net migrant with HbS trend lines, and given values for the HbS Index, DIV Index and Index of Change.

Supplementary Table 2 Net number of migrants and estimated net number of migrants with HbS (median, low and high estimates) per country based on the World Bank Bilateral Migration Database and HbS estimates published by Piel *et al* (2013).

Supplementary Material 1 Calculation of our Index of Change.

This Supplementary Material provides a detailed description of how we calculated our Index of Change summarised in Figure 5 in the main manuscript. The relationships between the different variables described below is summarised as a workflow diagram in Supplementary Figure 1. For illustrative purposes, we included at the end a couple of working examples for two countries: Nigeria and the United Kingdom.

1. MIG slope

MIG slope is the slope of the linear regression line summarising the temporal change over the period 1960-2000 in the relative net number of migrants for a given country. The slope was calculated as follows:

$$\alpha_{MIG} = \frac{\sum(x-\bar{x})(y-\bar{y})}{\sum(x-\bar{x})^2}$$

where α_{MIG} is the slope for the migrants; x is the year; y is the estimated net number of migrants with HbS; $\bar{x}$ is the average time point; $\bar{y}$ is the average relative number of net migrants.

2. HbS slope

HbS slope is the slope of the linear regression line summarising the temporal change over the period 1960-2000 in the estimated relative net number of migrants with HbS for a given country. The slope was calculated as follows:

$$\alpha_{HbS} = \frac{\sum(x-\bar{x})(z-\bar{z})}{\sum(x-\bar{x})^2}$$

where α_{HbS} is the slope for the migrant with HbS; x is the year; z is the estimated net number of migrants with HbS; $\bar{x}$ is the average time point; $\bar{z}$ is the average estimated net number of migrants with HbS.

3. HbS Index

This is an index defining the slope of the trend line in the estimated relative net numbers of migrants with HbS over the study period (1960-2000). The index uses three classes depending on the coefficient of the slope.

HbS slope	HbS Index
≤-2	-1
>-2 & <+2	0
≥+2	+1

4. Ratio (R)

The ratio, termed *R*, is calculated as the absolute value of HbS slope divided by MIG slope.

$$R = \left|\frac{HbS\ slope}{MIG\ slope}\right|$$

5. DIV Index

The DIV Index codes the divergence between the trend lines corresponding to linear regressions of the relative net number of migrants and the estimated relative net number of migrants with HbS, respectively. This measure is based on i) the value of the ratio, *R*; and ii) the sign of HbS slope and MIG slope. We categorised the ratio into five classes: high ratio for values >2, medium-high ratios for values between 1.35 and 2, average ratios for values between 0.66 and 1.35, medium-low ratios for values between 0.50 and 0.66, and finally low ratios for values below 0.50. When the slopes of the two trend lines had the same sign, we defined five classes of DIV Index based on the ratio between the two slopes (HbS slope/MIG slope). For negative slopes, a high ratio (*R*>2) was suggesting that the estimated relative net number of migrants with HbS was decreasing much slower over the study period than the relative net number of migrants with HbS for a given country, suggesting an improving situation in term of the burden of sickle cell in that country: the attributed DIV Index was therefore equal to -2; a low ratio (*R*≤0.50) was suggesting that the estimated relative net number of migrants with HbS was decreasing much faster over the study period than the relative net number of migrants with HbS for a given country, suggesting an improving situation in term of the burden of sickle cell in that country: the attributed DIV Index was therefore equal to +2. For positive slopes, a high ratio was suggesting that the estimated relative net number of migrants with HbS was increasing much faster over the study period than the relative net number of migrants with HbS for a given country, suggesting an improving situation in term of the burden of sickle cell in that country: the attributed DIV Index was therefore equal to +2; a low ratio was suggesting that the estimated relative net number of migrants with HbS was increasing much slower over the study period than the relative net number of migrants with HbS for a given country, suggesting an aggravating situation in term of the burden of sickle cell in that country: the attributed DIV Index was therefore equal to -2. The DIV Index was therefore equal to -2. When the slopes of the HbS and MIG trend lines were positive and negative, or negative and positive, respectively, it was not possible to define systematic rules because the divergence of the two trend lines depended on whether they were crossing each other or not, and at which point in time. As a result, we analysed the temporal plots on a case by case basis. A positive HbS slope combined with a negative MIG slope, observed for 35 countries, was suggesting of an aggravating situation in terms of the burden of sickle cell in the given country over the study period. Attributed values of the DIV index were therefore positive and ranged between 0 and +2. A negative HbS slope combined with a positive MIG slope, observed for only 5 countries, was suggesting of an improving situation in terms of the burden of sickle cell in the given country over the study period. Attributed values of the DIV index were therefore negative and ranged between -1 and -2. Full details of the rules used are summarised in Supplementary Figure 2. Theoretical illustrative examples of each combination are shown below. Red lines are showing the trends in the estimated relative net numbers of migrants with HbS. Blue lines are showing the trends in the relative net numbers of migrants. Grey discontinuous lines are showing the trends corresponding to the cut-off values used for the Ratio (*R*).

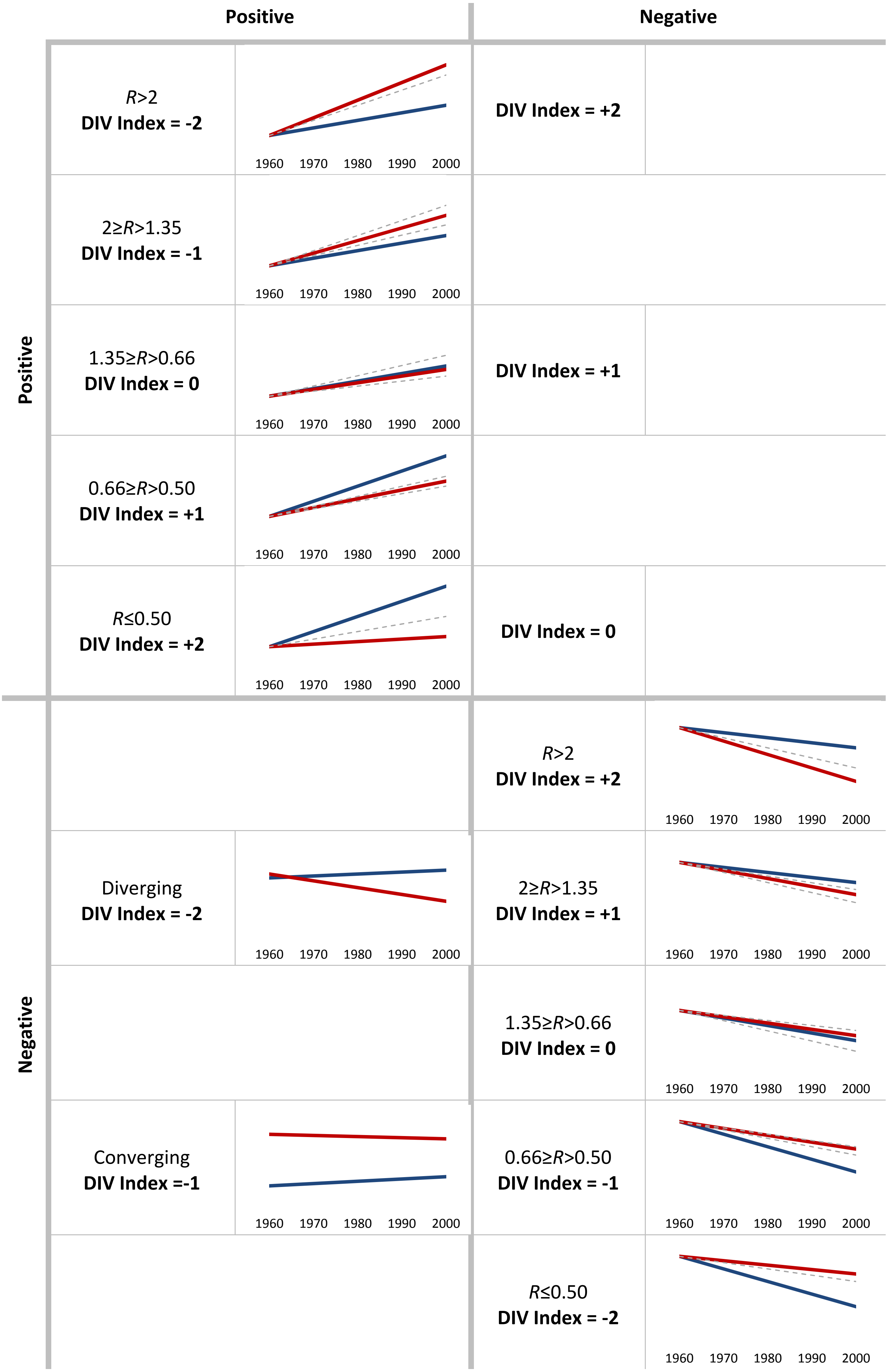
Positive
Negative
Positive
R>2
DIV Index = -2
DIV Index = +2
2≥R>1.35
DIV Index = -1
1.35≥R>0.66
DIV Index = 0
DIV Index = +1
0.66≥R>0.50
DIV Index = +1
R≤0.50
DIV Index = +2
DIV Index = 0
Negative
R>2
DIV Index = +2
Diverging
DIV Index = -2
2≥R>1.35
DIV Index = +1
1.35≥R>0.66
DIV Index = 0
Converging
DIV Index =-1
0.66≥R>0.50
DIV Index = -1
R≤0.50
DIV Index = -2
1960
1970
1980
1990
2000

6. Index of change

The Index of Change is calculated as the sum of the HbS Index and the DIV Index. The HbS Index values range between -1 and 1. The DIV Index values range between -2 and 2. As a result, the Index of Change values range between -3 and 3. The global map of our Index of Change is shown in Supplementary Figure 4.

$$Index\ of\ Change = HbS\ Index + DIV\ Index$$

7. Working examples:

Country	Nigeria	United Kingdom
	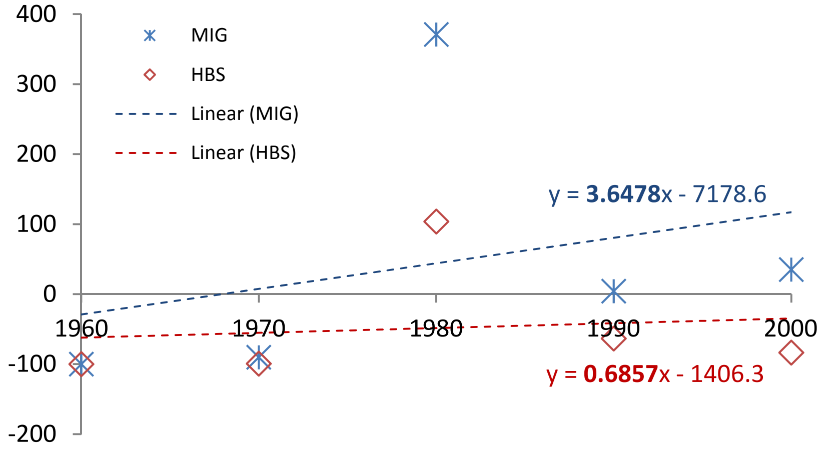	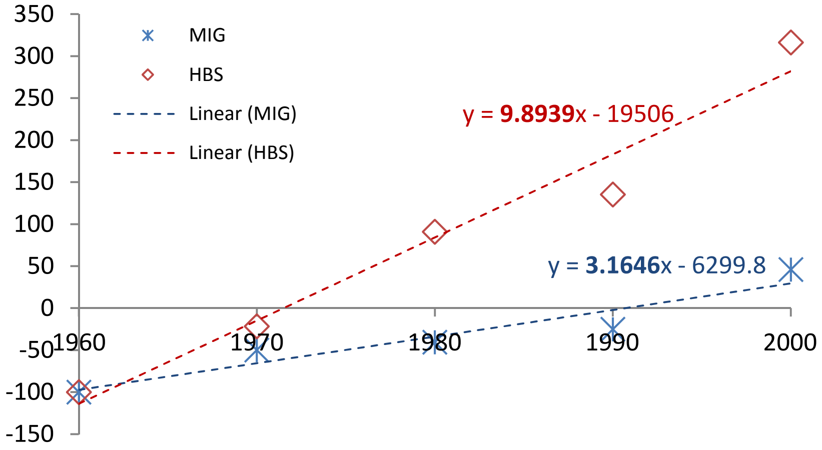
**MIG slope**	**3.65**	**3.16**
**HbS slope**	**0.69**	**9.89**
**Ratio (R)**	HbS slope/MIG slope<br>0.69/3.65 = **0.19**	HbS slope/MIG slope<br>9.89/3.16 = **3.13**
**DIV Index**	MIG slope = Positive; HbS slope = Positive; R≤0.50<br>**-2**	MIG slope = Positive; HbS slope = Positive; R>2.00<br>**2**
**HbS Index**	-2 < HbS slope < 2<br>**0**	HbS slope > 2<br>**1**
**Index of Change**	HbS Index + DIV Index<br>(0) + (-2) = **-2**	HbS Index + DIV Index<br>(1) + (2) = **3**

Supplementary Material 2 R code used to generate the flow maps. R is freely available from http://cran.r-project.org/bin/windows/base/

```
# Load require libraries

require(reshape)
require(igraph)
require(geosphere)
require(maptools)
require(sp)

# Eliminate not exit rows

DrawSingleLine <- function(x,district,idcol){

  inter <- gcIntermediate(c(district$X[district$District==x[1]], district$Y[district$District==x[1]]),
                          c(district$X[district$District==x[2]], district$Y[district$District==x[2]]),
                          n=50, addStartEnd=TRUE,sp=T,breakAtDateLine=T)
  inter@lines[[1]]@ID <-as.character(x[idcol])

  return(inter@lines[[1]])
}

Createline<-function(predictions,idcol2, Country){

  glist<- apply(predictions,1,DrawSingleLine,ididcol2,district=Country)
  glist<-SpatialLines(glist)
  glist<-SpatialLinesDataFrame(glist,predictions,match.ID=F)
  return(glist)

# Put them into SpatialLineDataframe
}
```

Supplementary Figures

Supplementary Figure 1 Flowchart summarising the approach used to calculate our Index of Change. Net numbers have been calculated as the difference between the number of immigrants and emigrants. Relative numbers reflect the migrant flows compared to their reference level in 1960 (100). MIG slope represents the slope of the linear regression fitted to the relative net number of migrants. HbS slope represents the slope of the linear regression fitted to the estimated relative net number of migrants with HbS. HbS Index represents a coded index of HbS slope in three classes. Ratio (R) is the ratio between MIG slope and HbS slope. DIV Index is the divergence index between the two trend lines fitted to the relative net numbers of migrants and migrants with HbS, respectively. DIV index values ranging from -2 to 2 were given based on the following cut-off values for *R*: 0.50, 0.60, 1.35 and 2.00. The Index of Change is the sum of HbS Index and DIV Index.

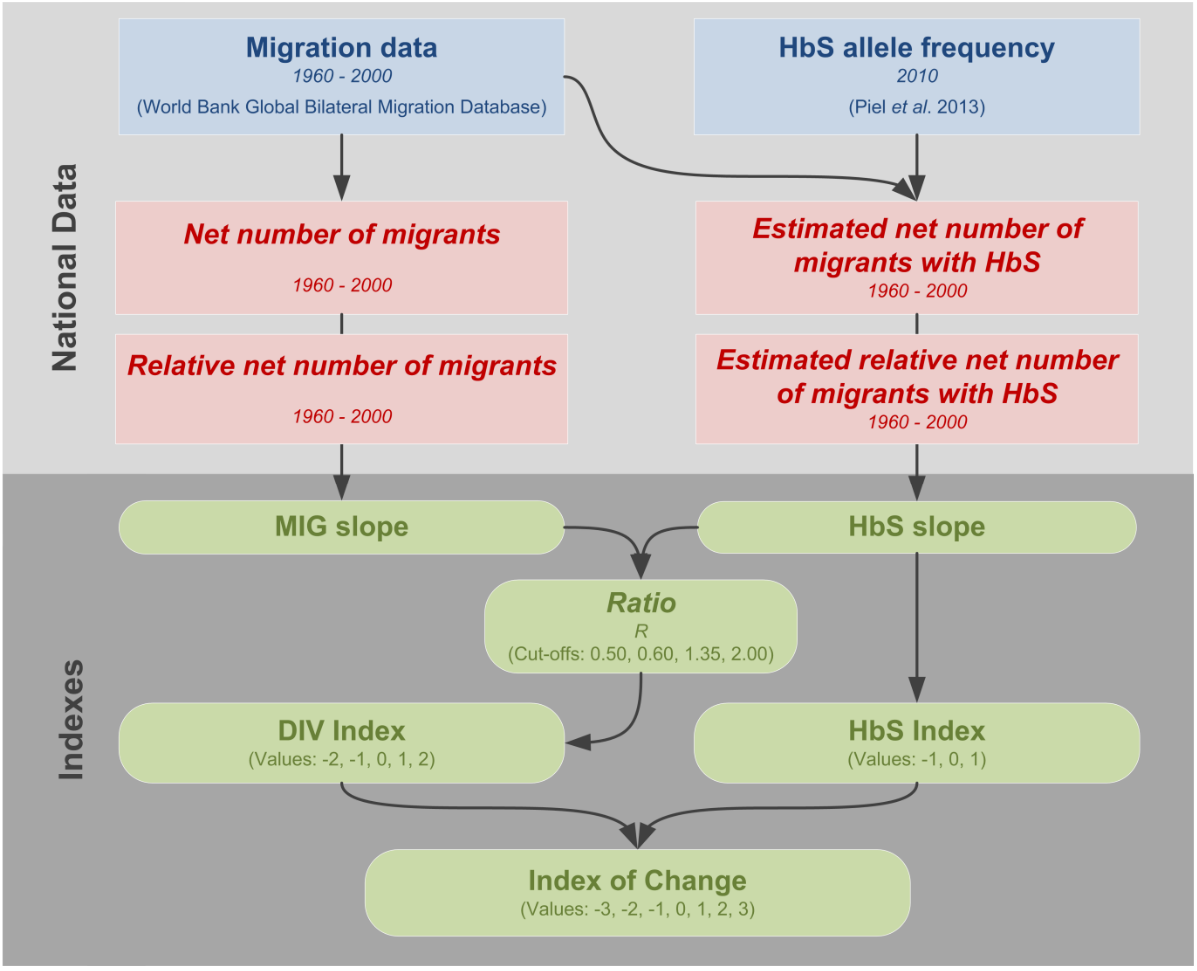

Supplementary Figure 2 Divergence index calculation rules. MIG slope (in blue) = slope of the trend in the number of overall net migrants between 1960 and 2000; HbS slope (in red) = slope of the trend in the estimated number of net migrants with HbS between 1960 and 2000; *R* = absolute ratio between HbS slope and MIG slope; *D* = absolute difference between HbS slope and MIG slope. n= number of countries falling within each of the quadrants. The values of the divergence index are shown in bold.

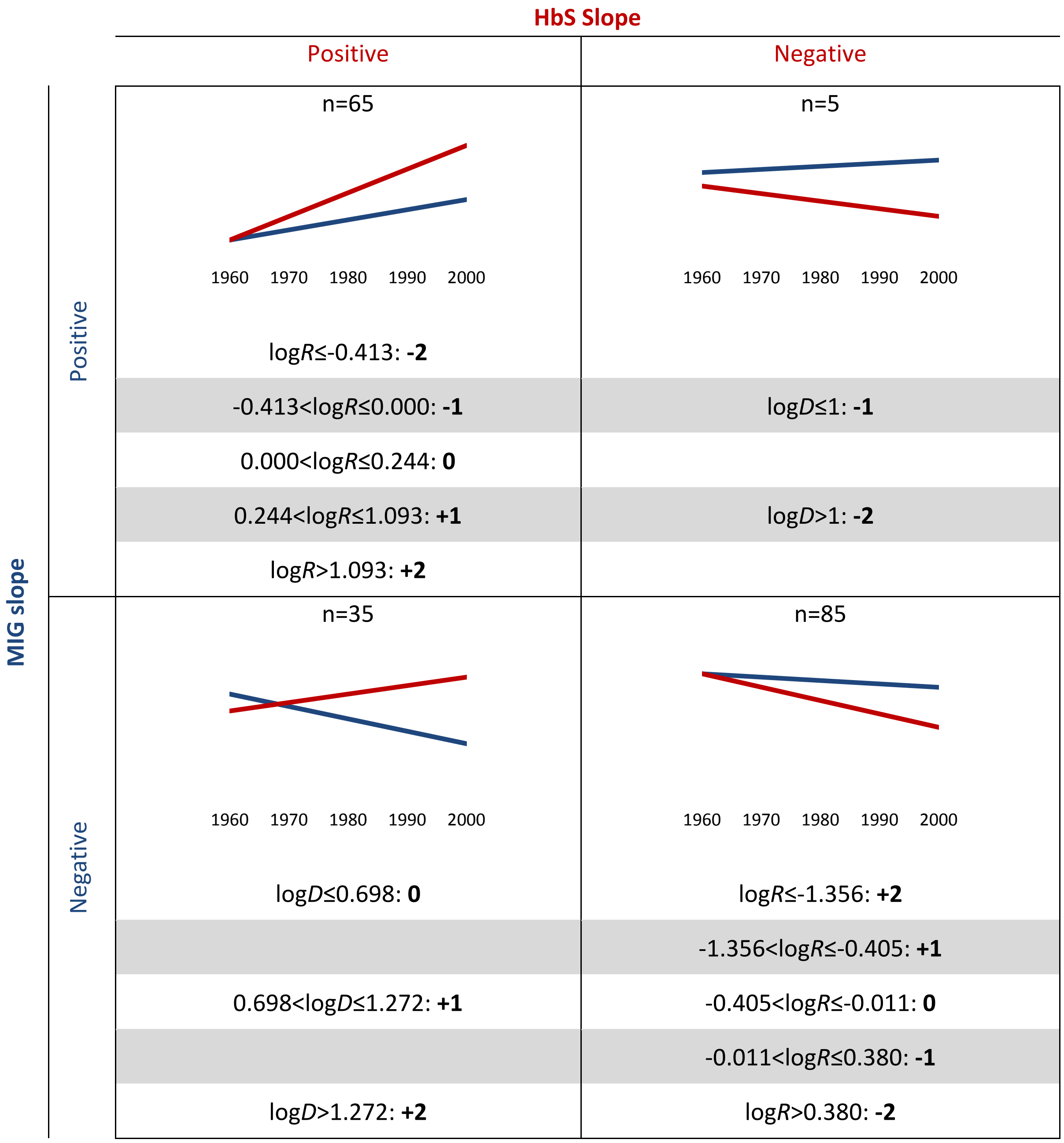

Supplementary Figure 3 Relative net numbers of migrants (blue line) and estimated relative net number of migrants with HbS (red line) compared to the 1960 level for all countries. Estimates based on the posterior median of HbS predictions published in Piel et al 2013. The red shaded area reflects the uncertainty (i.e. interquartile range) in the HbS predictions.

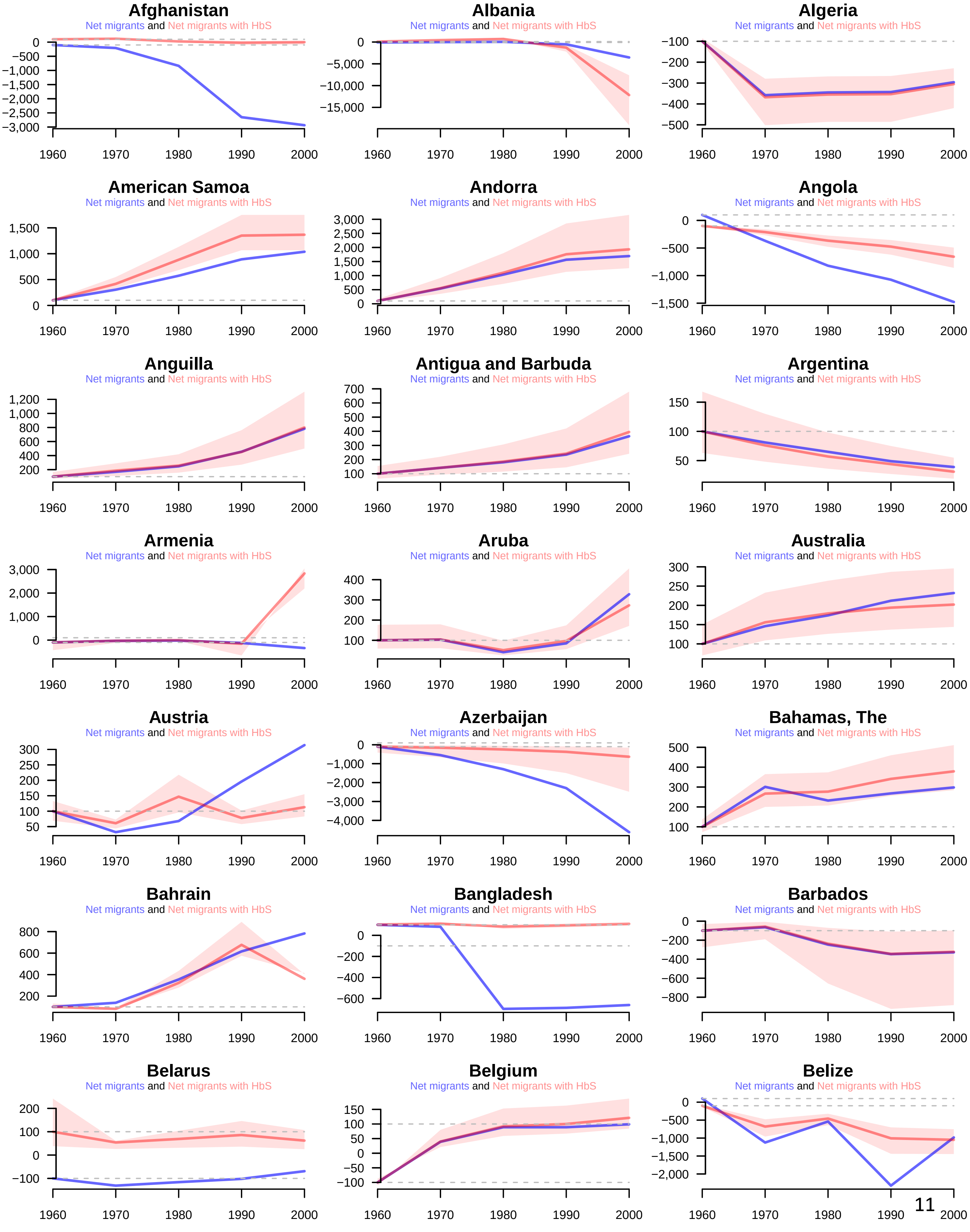

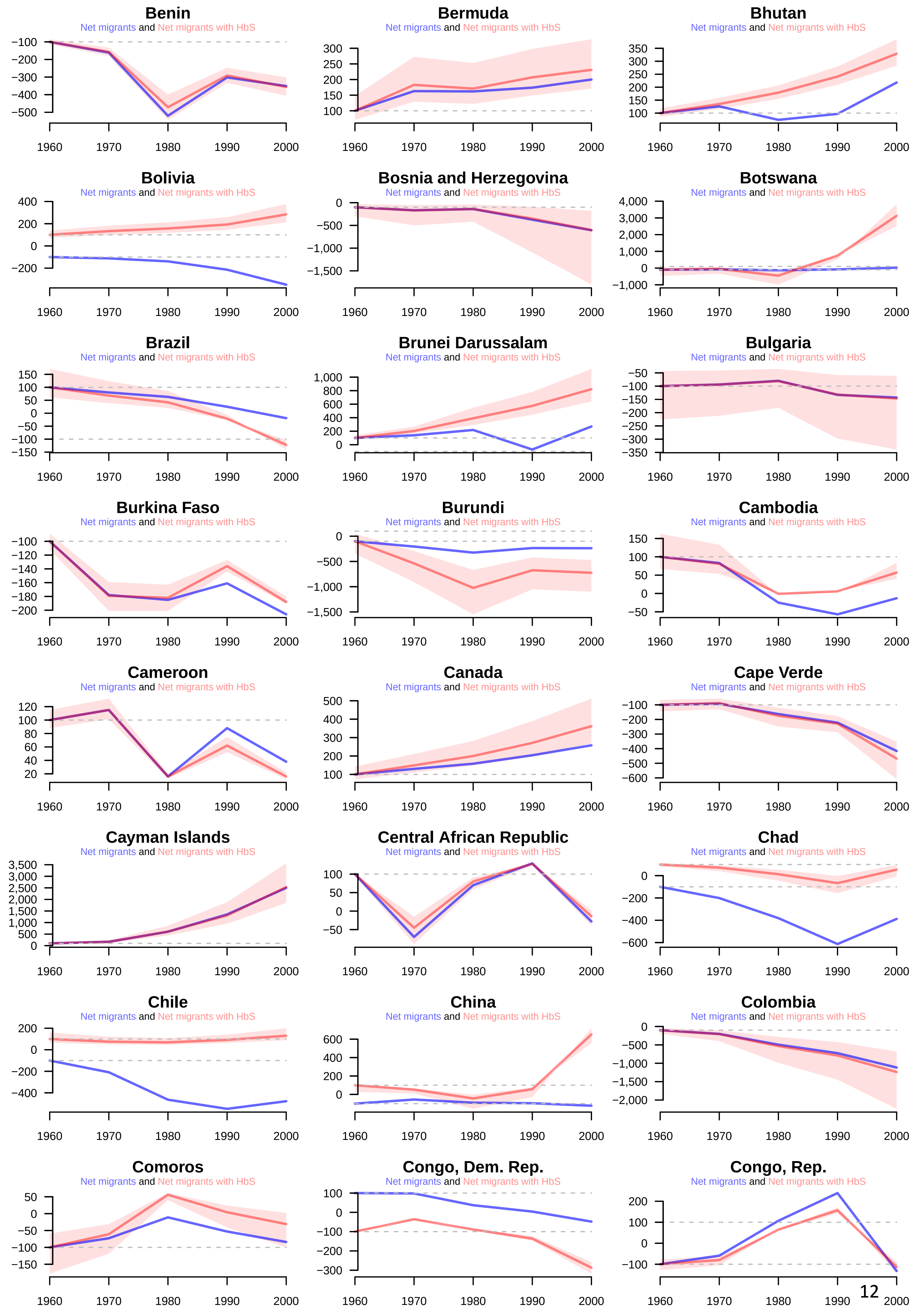
Benin
Net migrants and Net migrants with HbS
Bermuda
Net migrants and Net migrants with HbS
Bhutan
Net migrants and Net migrants with HbS
Bolivia
Net migrants and Net migrants with HbS
Bosnia and Herzegovina
Net migrants and Net migrants with HbS
Botswana
Net migrants and Net migrants with HbS
Brazil
Net migrants and Net migrants with HbS
Brunei Darussalam
Net migrants and Net migrants with HbS
Bulgaria
Net migrants and Net migrants with HbS
Burkina Faso
Net migrants and Net migrants with HbS
Burundi
Net migrants and Net migrants with HbS
Cambodia
Net migrants and Net migrants with HbS
Cameroon
Net migrants and Net migrants with HbS
Canada
Net migrants and Net migrants with HbS
Cape Verde
Net migrants and Net migrants with HbS
Cayman Islands
Net migrants and Net migrants with HbS
Central African Republic
Net migrants and Net migrants with HbS
Chad
Net migrants and Net migrants with HbS
Chile
Net migrants and Net migrants with HbS
China
Net migrants and Net migrants with HbS
Colombia
Net migrants and Net migrants with HbS
Comoros
Net migrants and Net migrants with HbS
Congo, Dem. Rep.
Net migrants and Net migrants with HbS
Congo, Rep.
Net migrants and Net migrants with HbS
1960
1970
1980
1990
2000

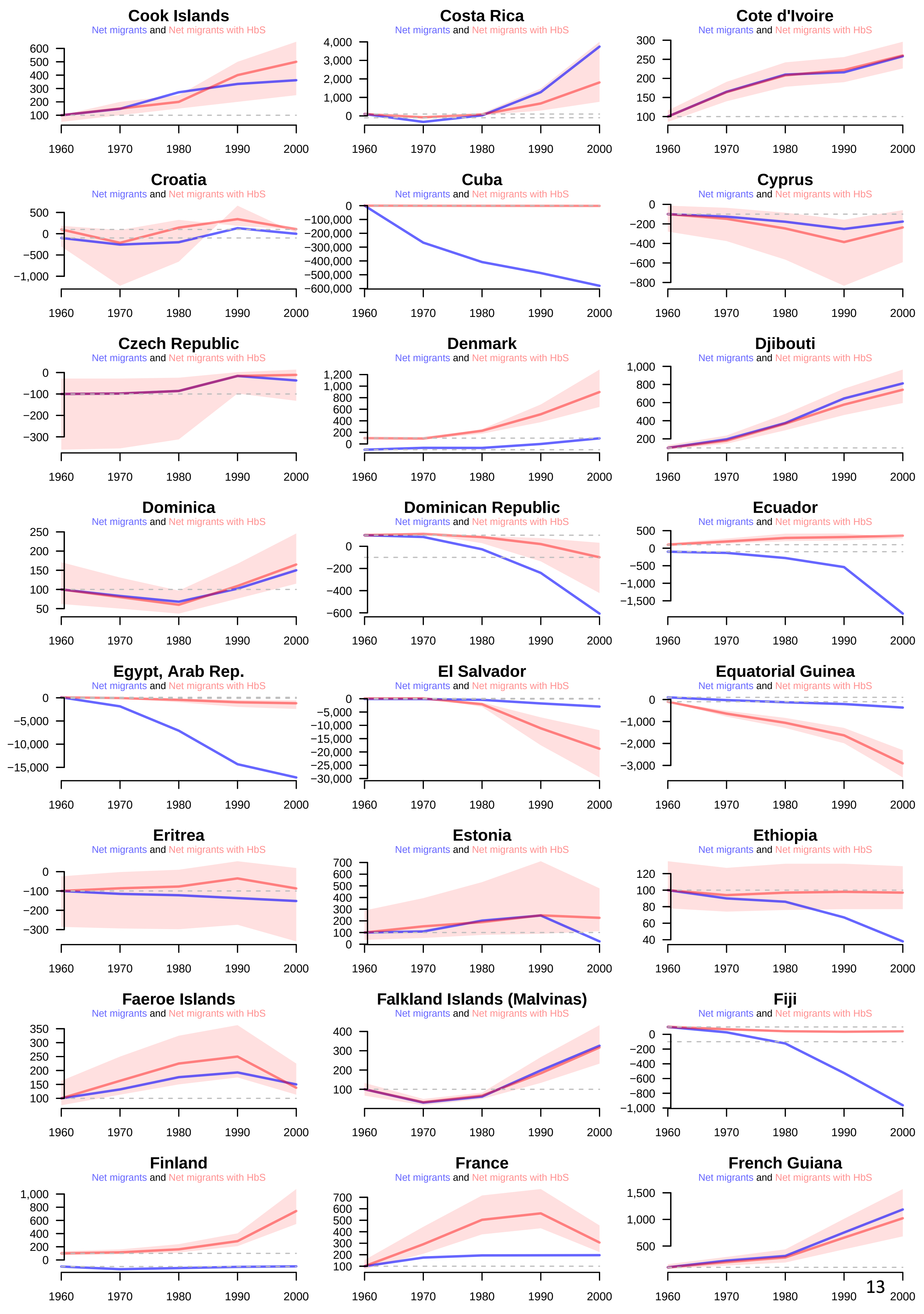
Cook Islands
Net migrants and Net migrants with HbS
Costa Rica
Net migrants and Net migrants with HbS
Cote d'Ivoire
Net migrants and Net migrants with HbS
Croatia
Net migrants and Net migrants with HbS
Cuba
Net migrants and Net migrants with HbS
Cyprus
Net migrants and Net migrants with HbS
Czech Republic
Net migrants and Net migrants with HbS
Denmark
Net migrants and Net migrants with HbS
Djibouti
Net migrants and Net migrants with HbS
Dominica
Net migrants and Net migrants with HbS
Dominican Republic
Net migrants and Net migrants with HbS
Ecuador
Net migrants and Net migrants with HbS
Egypt, Arab Rep.
Net migrants and Net migrants with HbS
El Salvador
Net migrants and Net migrants with HbS
Equatorial Guinea
Net migrants and Net migrants with HbS
Eritrea
Net migrants and Net migrants with HbS
Estonia
Net migrants and Net migrants with HbS
Ethiopia
Net migrants and Net migrants with HbS
Faeroe Islands
Net migrants and Net migrants with HbS
Falkland Islands (Malvinas)
Net migrants and Net migrants with HbS
Fiji
Net migrants and Net migrants with HbS
Finland
Net migrants and Net migrants with HbS
France
Net migrants and Net migrants with HbS
French Guiana
Net migrants and Net migrants with HbS
1960
1970
1980
1990
2000

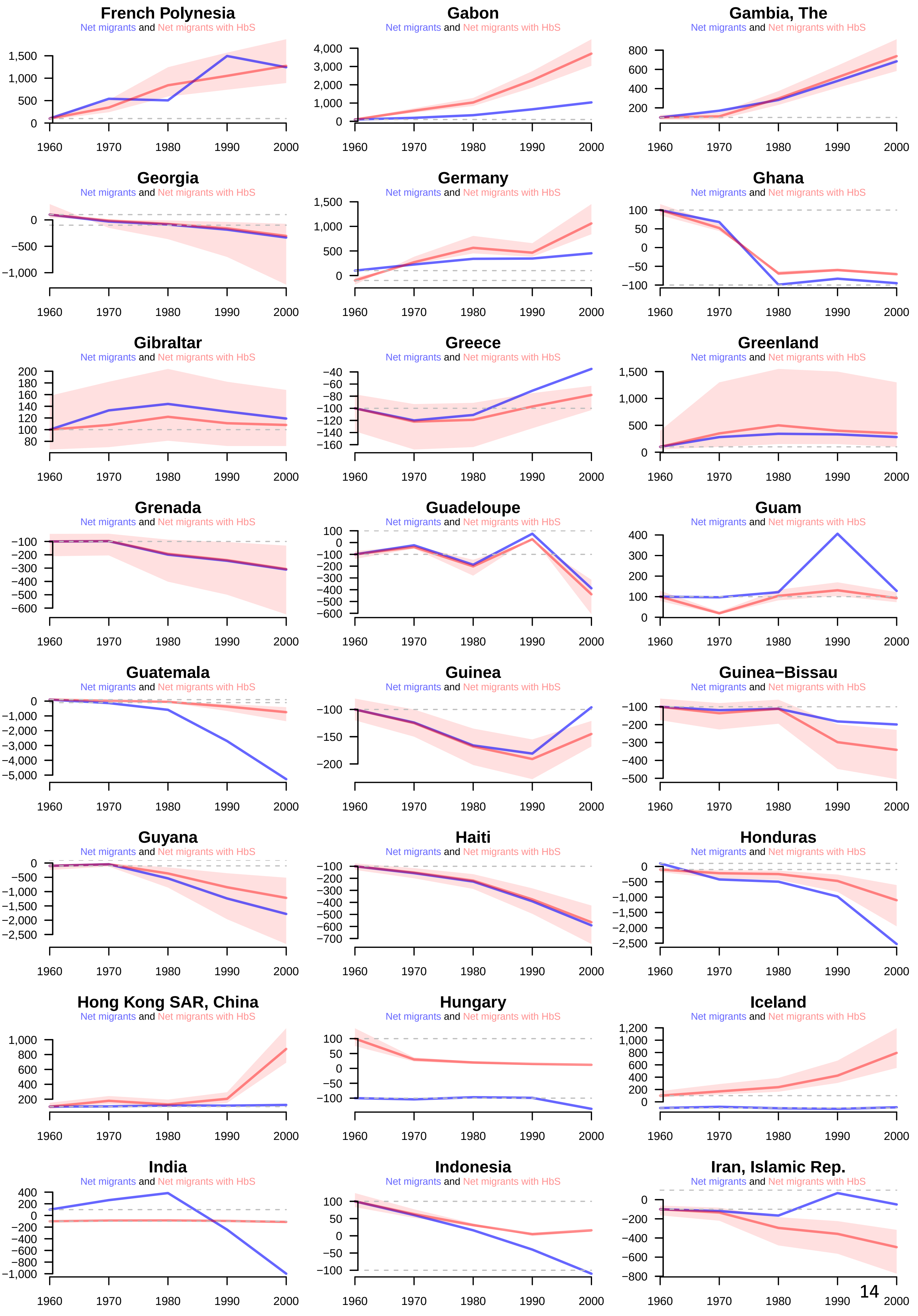
French Polynesia
Net migrants and Net migrants with HbS
Gabon
Net migrants and Net migrants with HbS
Gambia, The
Net migrants and Net migrants with HbS
Georgia
Net migrants and Net migrants with HbS
Germany
Net migrants and Net migrants with HbS
Ghana
Net migrants and Net migrants with HbS
Gibraltar
Net migrants and Net migrants with HbS
Greece
Net migrants and Net migrants with HbS
Greenland
Net migrants and Net migrants with HbS
Grenada
Net migrants and Net migrants with HbS
Guadeloupe
Net migrants and Net migrants with HbS
Guam
Net migrants and Net migrants with HbS
Guatemala
Net migrants and Net migrants with HbS
Guinea
Net migrants and Net migrants with HbS
Guinea−Bissau
Net migrants and Net migrants with HbS
Guyana
Net migrants and Net migrants with HbS
Haiti
Net migrants and Net migrants with HbS
Honduras
Net migrants and Net migrants with HbS
Hong Kong SAR, China
Net migrants and Net migrants with HbS
Hungary
Net migrants and Net migrants with HbS
Iceland
Net migrants and Net migrants with HbS
India
Net migrants and Net migrants with HbS
Indonesia
Net migrants and Net migrants with HbS
Iran, Islamic Rep.
Net migrants and Net migrants with HbS

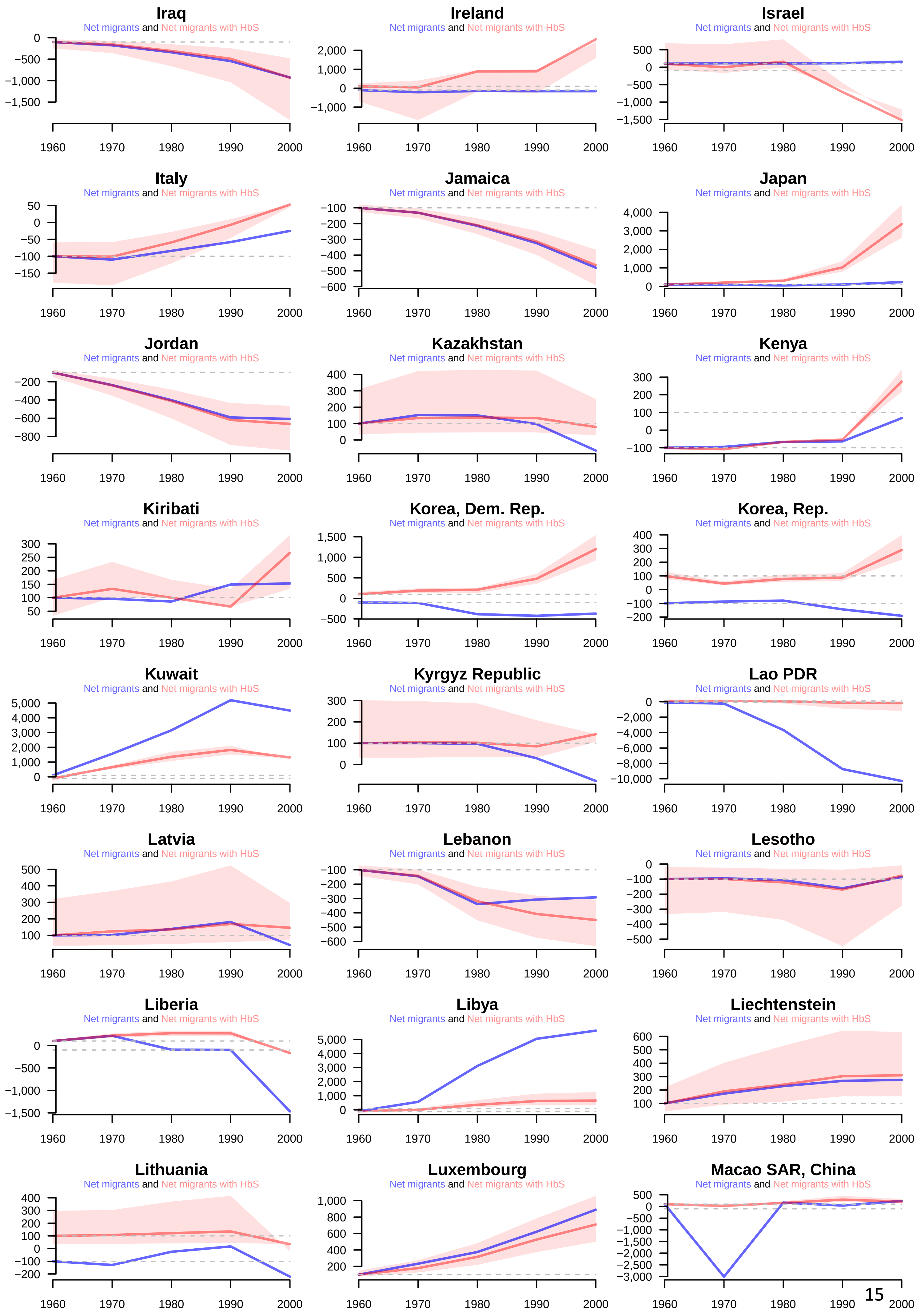
Iraq
Net migrants and Net migrants with HbS
Ireland
Net migrants and Net migrants with HbS
Israel
Net migrants and Net migrants with HbS
Italy
Net migrants and Net migrants with HbS
Jamaica
Net migrants and Net migrants with HbS
Japan
Net migrants and Net migrants with HbS
Jordan
Net migrants and Net migrants with HbS
Kazakhstan
Net migrants and Net migrants with HbS
Kenya
Net migrants and Net migrants with HbS
Kiribati
Net migrants and Net migrants with HbS
Korea, Dem. Rep.
Net migrants and Net migrants with HbS
Korea, Rep.
Net migrants and Net migrants with HbS
Kuwait
Net migrants and Net migrants with HbS
Kyrgyz Republic
Net migrants and Net migrants with HbS
Lao PDR
Net migrants and Net migrants with HbS
Latvia
Net migrants and Net migrants with HbS
Lebanon
Net migrants and Net migrants with HbS
Lesotho
Net migrants and Net migrants with HbS
Liberia
Net migrants and Net migrants with HbS
Libya
Net migrants and Net migrants with HbS
Liechtenstein
Net migrants and Net migrants with HbS
Lithuania
Net migrants and Net migrants with HbS
Luxembourg
Net migrants and Net migrants with HbS
Macao SAR, China
Net migrants and Net migrants with HbS

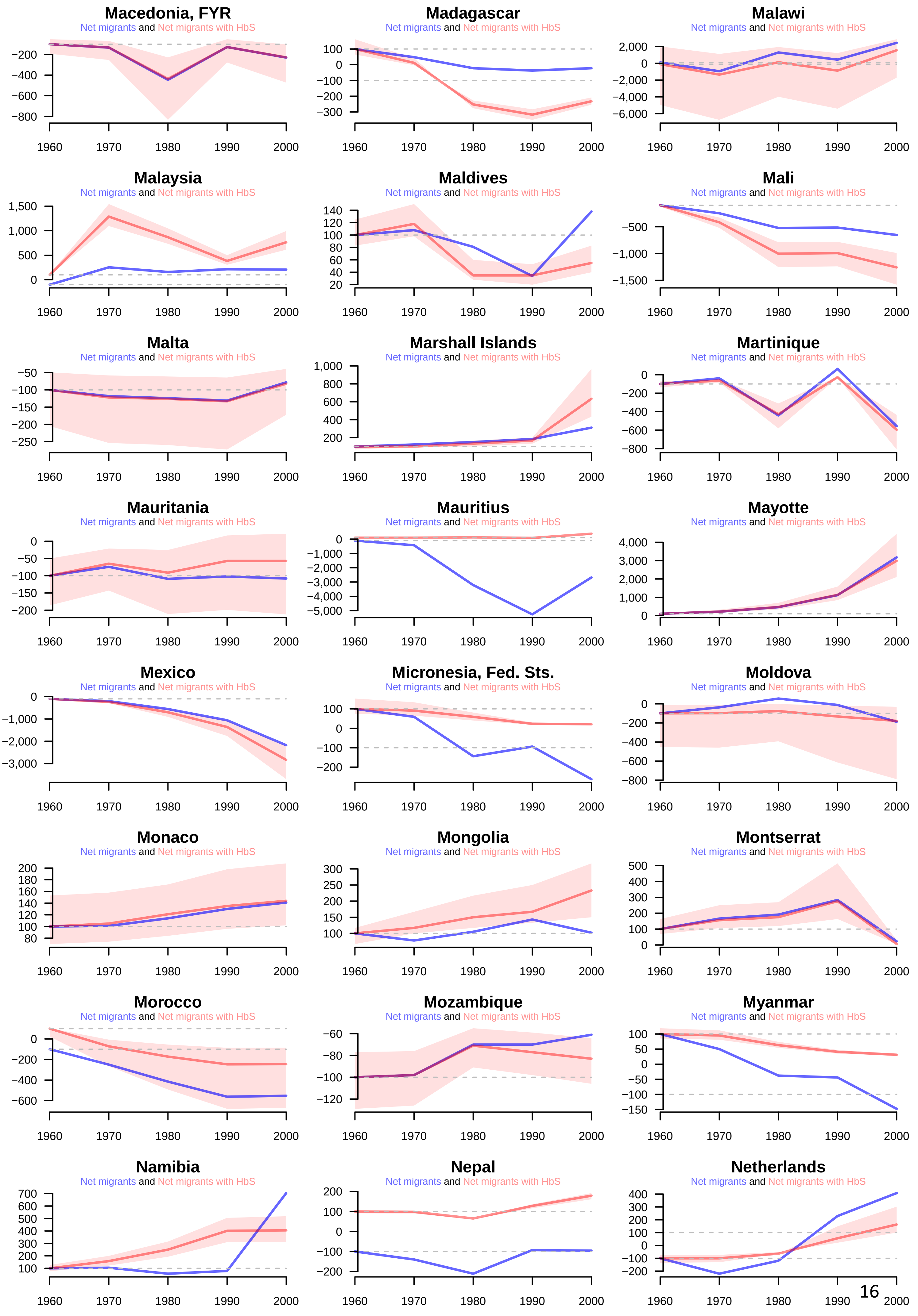
Macedonia, FYR
Net migrants and Net migrants with HbS
Madagascar
Net migrants and Net migrants with HbS
Malawi
Net migrants and Net migrants with HbS
Malaysia
Net migrants and Net migrants with HbS
Maldives
Net migrants and Net migrants with HbS
Mali
Net migrants and Net migrants with HbS
Malta
Net migrants and Net migrants with HbS
Marshall Islands
Net migrants and Net migrants with HbS
Martinique
Net migrants and Net migrants with HbS
Mauritania
Net migrants and Net migrants with HbS
Mauritius
Net migrants and Net migrants with HbS
Mayotte
Net migrants and Net migrants with HbS
Mexico
Net migrants and Net migrants with HbS
Micronesia, Fed. Sts.
Net migrants and Net migrants with HbS
Moldova
Net migrants and Net migrants with HbS
Monaco
Net migrants and Net migrants with HbS
Mongolia
Net migrants and Net migrants with HbS
Montserrat
Net migrants and Net migrants with HbS
Morocco
Net migrants and Net migrants with HbS
Mozambique
Net migrants and Net migrants with HbS
Myanmar
Net migrants and Net migrants with HbS
Namibia
Net migrants and Net migrants with HbS
Nepal
Net migrants and Net migrants with HbS
Netherlands
Net migrants and Net migrants with HbS
1960
1970
1980
1990
2000

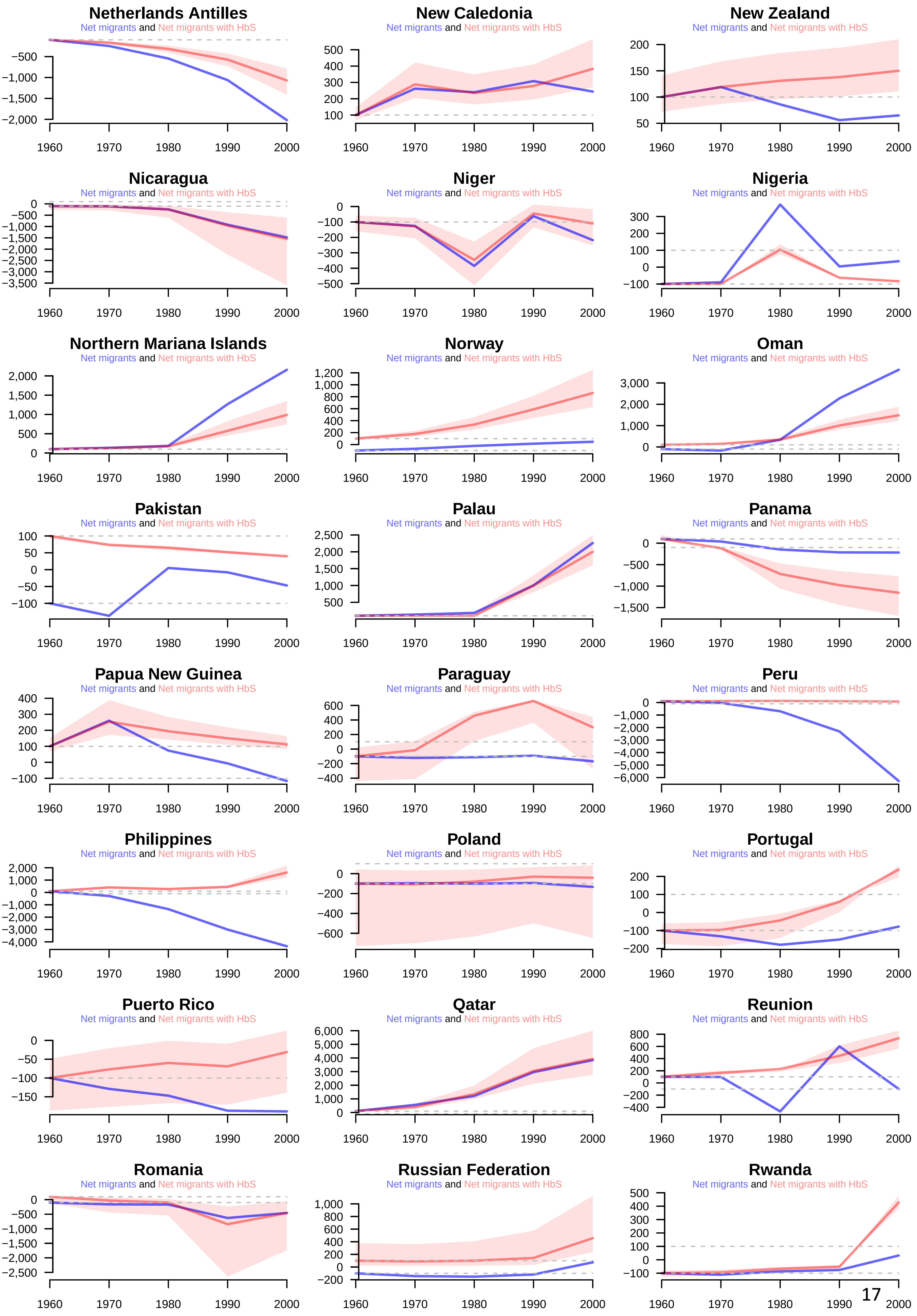
Netherlands Antilles
Net migrants and Net migrants with HbS
New Caledonia
Net migrants and Net migrants with HbS
New Zealand
Net migrants and Net migrants with HbS
Nicaragua
Net migrants and Net migrants with HbS
Niger
Net migrants and Net migrants with HbS
Nigeria
Net migrants and Net migrants with HbS
Northern Mariana Islands
Net migrants and Net migrants with HbS
Norway
Net migrants and Net migrants with HbS
Oman
Net migrants and Net migrants with HbS
Pakistan
Net migrants and Net migrants with HbS
Palau
Net migrants and Net migrants with HbS
Panama
Net migrants and Net migrants with HbS
Papua New Guinea
Net migrants and Net migrants with HbS
Paraguay
Net migrants and Net migrants with HbS
Peru
Net migrants and Net migrants with HbS
Philippines
Net migrants and Net migrants with HbS
Poland
Net migrants and Net migrants with HbS
Portugal
Net migrants and Net migrants with HbS
Puerto Rico
Net migrants and Net migrants with HbS
Qatar
Net migrants and Net migrants with HbS
Reunion
Net migrants and Net migrants with HbS
Romania
Net migrants and Net migrants with HbS
Russian Federation
Net migrants and Net migrants with HbS
Rwanda
Net migrants and Net migrants with HbS

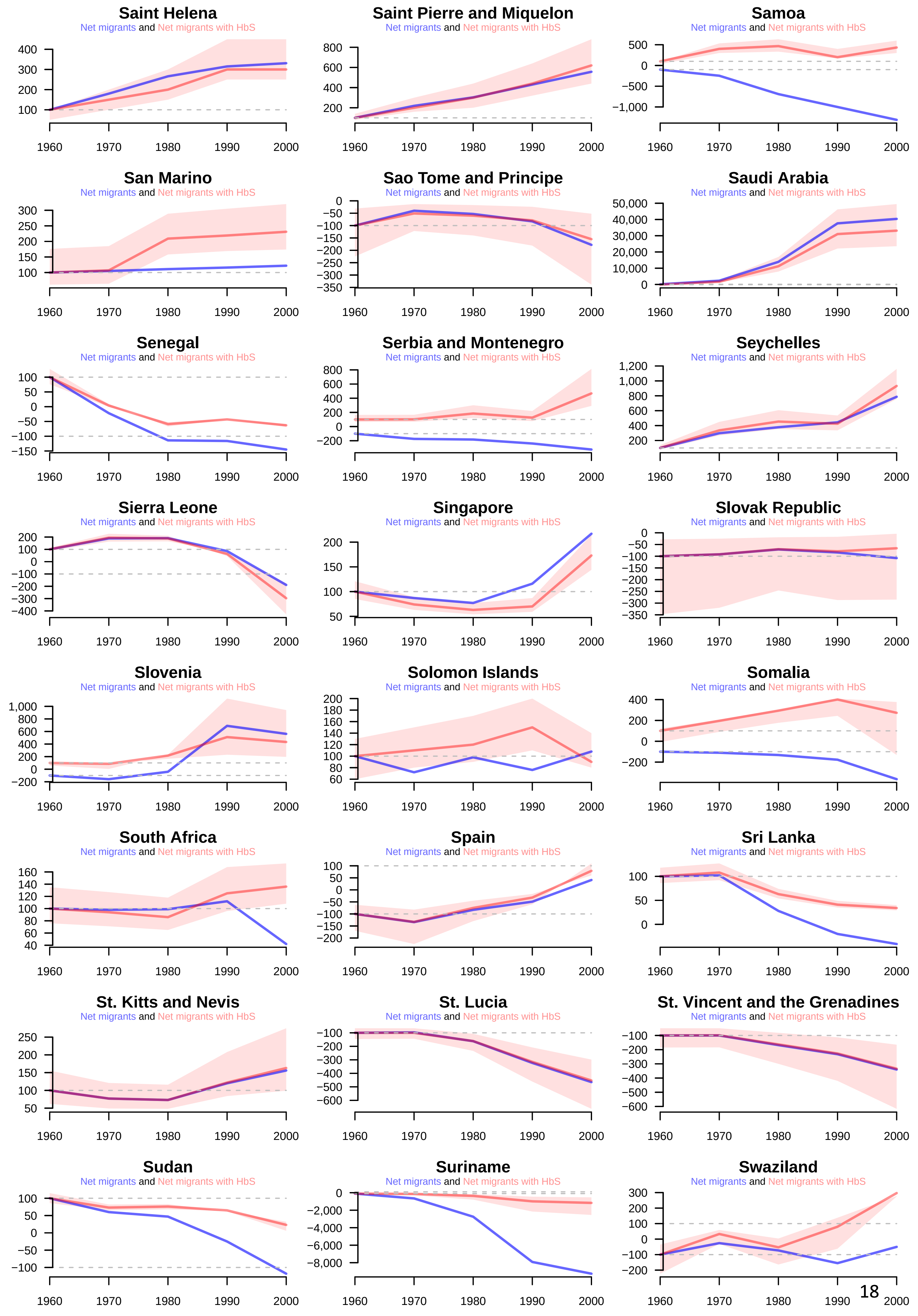
Saint Helena
Net migrants and Net migrants with HbS
Saint Pierre and Miquelon
Net migrants and Net migrants with HbS
Samoa
Net migrants and Net migrants with HbS
San Marino
Net migrants and Net migrants with HbS
Sao Tome and Principe
Net migrants and Net migrants with HbS
Saudi Arabia
Net migrants and Net migrants with HbS
Senegal
Net migrants and Net migrants with HbS
Serbia and Montenegro
Net migrants and Net migrants with HbS
Seychelles
Net migrants and Net migrants with HbS
Sierra Leone
Net migrants and Net migrants with HbS
Singapore
Net migrants and Net migrants with HbS
Slovak Republic
Net migrants and Net migrants with HbS
Slovenia
Net migrants and Net migrants with HbS
Solomon Islands
Net migrants and Net migrants with HbS
Somalia
Net migrants and Net migrants with HbS
South Africa
Net migrants and Net migrants with HbS
Spain
Net migrants and Net migrants with HbS
Sri Lanka
Net migrants and Net migrants with HbS
St. Kitts and Nevis
Net migrants and Net migrants with HbS
St. Lucia
Net migrants and Net migrants with HbS
St. Vincent and the Grenadines
Net migrants and Net migrants with HbS
Sudan
Net migrants and Net migrants with HbS
Suriname
Net migrants and Net migrants with HbS
Swaziland
Net migrants and Net migrants with HbS
1960
1970
1980
1990
2000

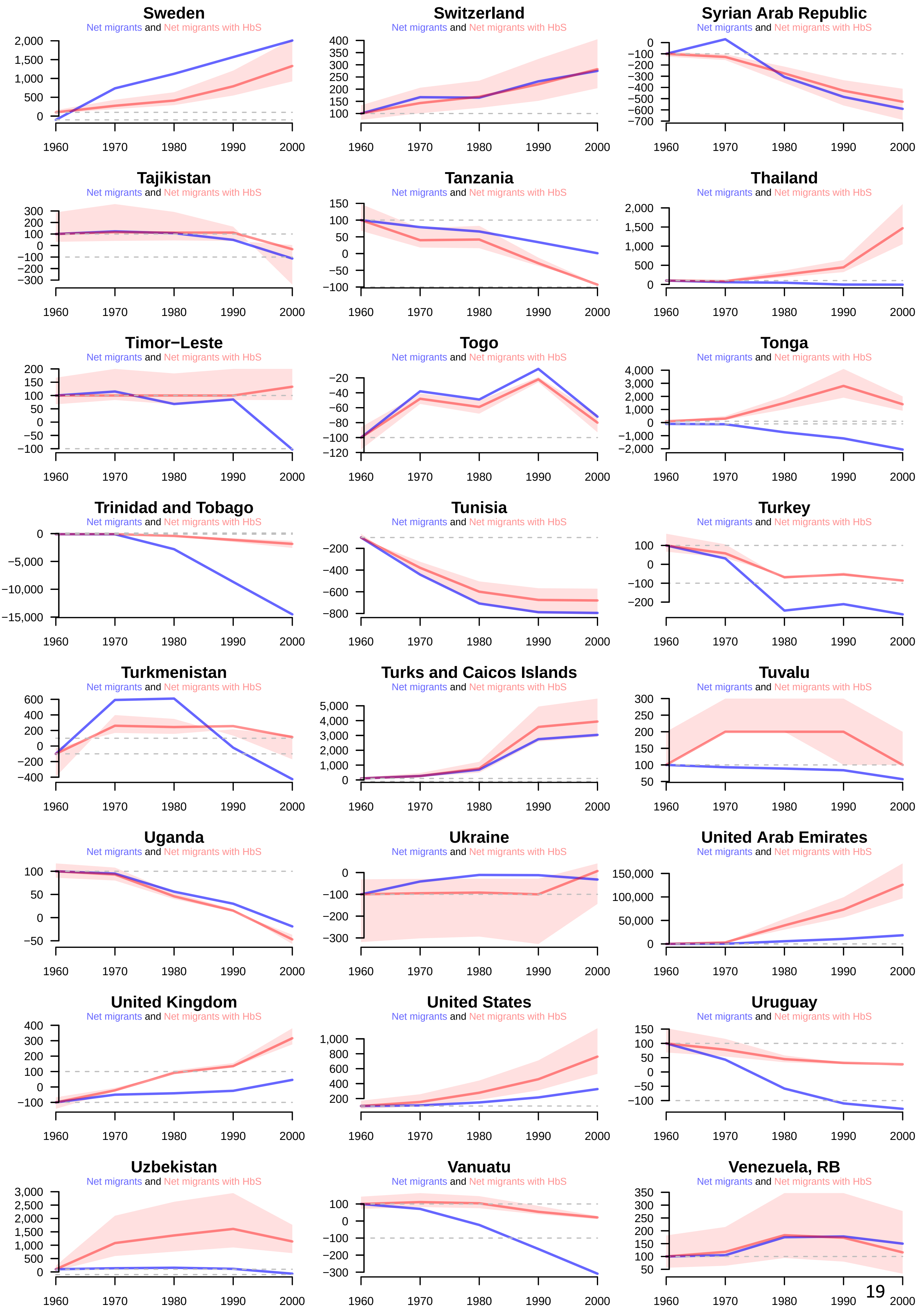
Sweden
Net migrants and Net migrants with HbS
Switzerland
Net migrants and Net migrants with HbS
Syrian Arab Republic
Net migrants and Net migrants with HbS
Tajikistan
Net migrants and Net migrants with HbS
Tanzania
Net migrants and Net migrants with HbS
Thailand
Net migrants and Net migrants with HbS
Timor−Leste
Net migrants and Net migrants with HbS
Togo
Net migrants and Net migrants with HbS
Tonga
Net migrants and Net migrants with HbS
Trinidad and Tobago
Net migrants and Net migrants with HbS
Tunisia
Net migrants and Net migrants with HbS
Turkey
Net migrants and Net migrants with HbS
Turkmenistan
Net migrants and Net migrants with HbS
Turks and Caicos Islands
Net migrants and Net migrants with HbS
Tuvalu
Net migrants and Net migrants with HbS
Uganda
Net migrants and Net migrants with HbS
Ukraine
Net migrants and Net migrants with HbS
United Arab Emirates
Net migrants and Net migrants with HbS
United Kingdom
Net migrants and Net migrants with HbS
United States
Net migrants and Net migrants with HbS
Uruguay
Net migrants and Net migrants with HbS
Uzbekistan
Net migrants and Net migrants with HbS
Vanuatu
Net migrants and Net migrants with HbS
Venezuela, RB
Net migrants and Net migrants with HbS

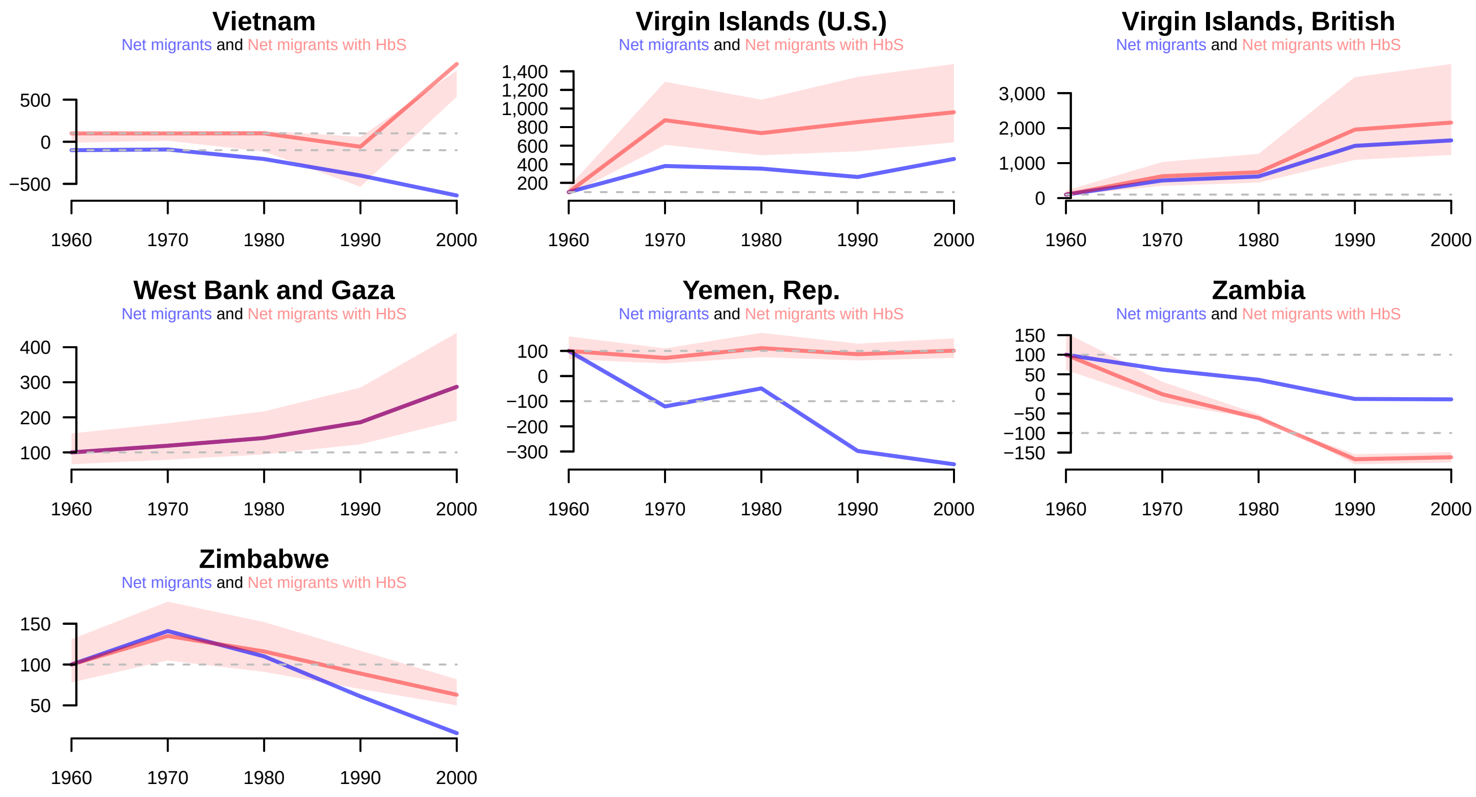
Vietnam
Net migrants and Net migrants with HbS
500
0
−500
1960
1970
1980
1990
2000
Virgin Islands (U.S.)
Net migrants and Net migrants with HbS
1,400
1,200
1,000
800
600
400
200
1960
1970
1980
1990
2000
Virgin Islands, British
Net migrants and Net migrants with HbS
3,000
2,000
1,000
0
1960
1970
1980
1990
2000
West Bank and Gaza
Net migrants and Net migrants with HbS
400
300
200
100
1960
1970
1980
1990
2000
Yemen, Rep.
Net migrants and Net migrants with HbS
100
0
−100
−200
−300
1960
1970
1980
1990
2000
Zambia
Net migrants and Net migrants with HbS
150
100
50
0
−50
−100
−150
1960
1970
1980
1990
2000
Zimbabwe
Net migrants and Net migrants with HbS
150
100
50
1960
1970
1980
1990
2000

Supplementary Figure 4 Map of our Index of Change. Classification of countries based on absolute trends in the net number of migrants with HbS during the study period (1960-2000) and relative trends compared to the overall net number of migrants. The index is based on the slope of the trend line for net HbS migrants (HbS Index) and the divergence between the trend lines for net HbS migrants and net overall migrants (DIV Index). A positive Index, shown in red, suggests that the estimated net flux of migrants with HbS has proportionally increased over the study period and that implementing specific interventions for the prevention of sickle cell disease should likely be considered. A negative Index, shown in blue, suggests that the estimated net flux of migrants with HbS has proportionally decreased over the study period and that specific interventions for the prevention of sickle cell disease are unlikely to be a health priority.

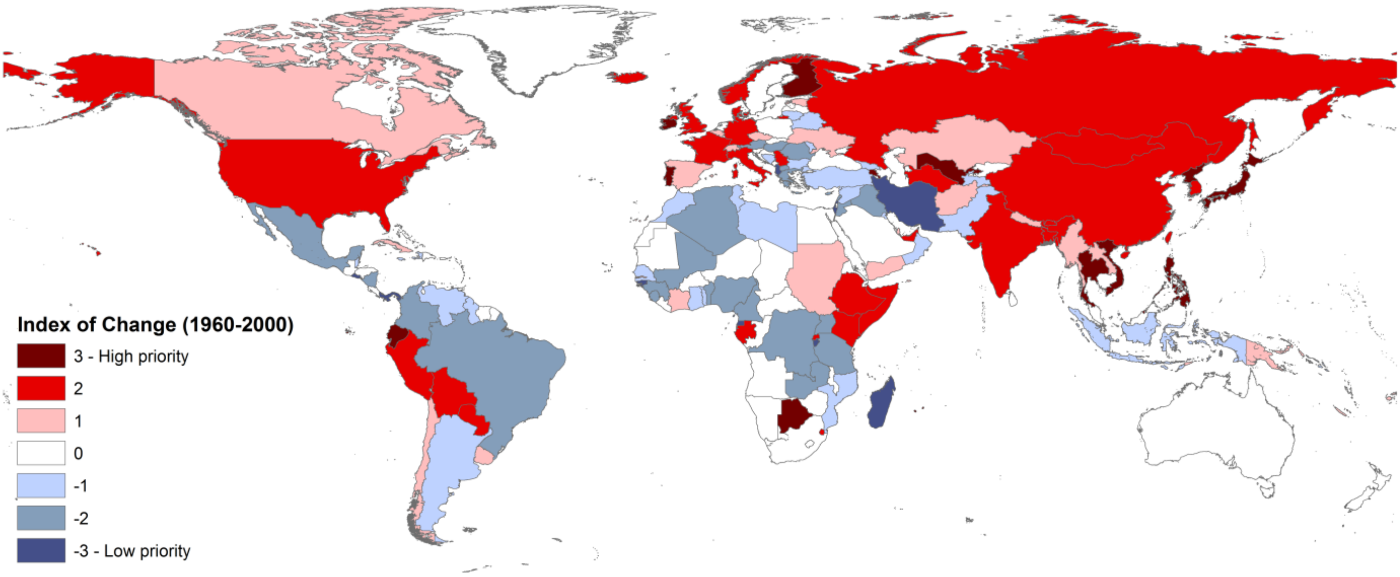

Supplementary Tables

Supplementary Table 1. Slope coefficients for the overall net migrant (MIG) and net migrant with HbS trend lines, and given values for the HbS Index, DIV Index and Index of Change. See Supplementary Material A for details.

Country	HbS slope	MIG slope	Group	Ratio (R)/Difference (D)	LogR/LogD	HbS Index	DIV Index	Index of Change
Aruba	3.390	4.371	+/+	0.775	-0.110	1	-1	0
Australia	2.430	3.301	+/+	0.736	-0.133	1	-1	0
Austria	0.420	5.927	+/+	0.071	-1.149	0	-2	-2
Bahrain	11.195	18.448	+/+	0.607	-0.217	1	-1	0
Belgium	5.026	4.487	+/+	1.120	0.049	1	0	1
Bhutan	5.637	2.062	+/+	2.734	0.437	1	1	2
Botswana	72.465	2.452	+/+	29.552	1.471	1	2	3
Brunei Darussalam	18.156	1.290	+/+	14.073	1.148	1	2	3
Canada	6.457	3.902	+/+	1.655	0.219	1	0	1
Comoros	2.031	0.514	+/+	3.954	0.597	1	1	2
Congo, Rep.	2.112	2.349	+/+	0.899	-0.046	1	-1	0
Costa Rica	41.633	89.009	+/+	0.468	-0.330	1	-1	0
Cote d'Ivoire	3.777	3.670	+/+	1.029	0.013	1	0	1
Croatia	5.735	5.822	+/+	0.985	-0.007	1	-1	0
Czech Republic	2.603	2.067	+/+	1.259	0.100	1	0	1
Denmark	20.130	4.603	+/+	4.373	0.641	1	1	2
Djibouti	16.818	18.765	+/+	0.896	-0.048	1	-1	0
Finland	14.547	0.408	+/+	35.622	1.552	1	2	3
France	6.795	2.113	+/+	3.217	0.507	1	1	2
French Guiana	23.048	27.021	+/+	0.853	-0.069	1	-1	0
French Polynesia	30.563	32.417	+/+	0.943	-0.026	1	-1	0
Gabon	88.768	23.387	+/+	3.796	0.579	1	1	2
Gambia, The	16.834	14.781	+/+	1.139	0.056	1	0	1
Germany	25.151	8.273	+/+	3.040	0.483	1	1	2
Greece	0.688	1.796	+/+	0.383	-0.417	0	-2	-2
Guam	0.978	3.649	+/+	0.268	-0.572	0	-2	-2
Hong Kong SAR, China	15.763	0.561	+/+	28.109	1.449	1	2	3
Italy	4.007	2.013	+/+	1.991	0.299	1	1	2
Japan	73.599	2.793	+/+	26.355	1.421	1	2	3
Kenya	8.041	3.671	+/+	2.190	0.341	1	1	2
Kuwait	40.024	124.065	+/+	0.323	-0.491	1	-2	-1
Libya	21.390	159.211	+/+	0.134	-0.872	1	-2	-1
Luxembourg	15.681	19.707	+/+	0.796	-0.099	1	-1	0
Macao SAR, China	4.846	33.245	+/+	0.146	-0.836	1	-2	-1
Malawi	37.917	60.735	+/+	0.624	-0.205	1	-1	0
Malaysia	4.251	5.748	+/+	0.740	-0.131	1	-1	0
Malta	0.247	0.303	+/+	0.814	-0.090	0	-1	-1
Mayotte	66.930	70.556	+/+	0.949	-0.023	1	-1	0
Mongolia	3.167	0.689	+/+	4.594	0.662	1	1	2
Mozambique	0.550	1.064	+/+	0.517	-0.287	0	-1	-1
Namibia	8.529	11.816	+/+	0.722	-0.142	1	-1	0
Nepal	1.874	0.549	+/+	3.411	0.533	0	1	1
Netherlands	6.818	14.642	+/+	0.466	-0.332	1	-1	0
New Caledonia	5.549	3.348	+/+	1.658	0.219	1	0	1
Nigeria	0.686	3.648	+/+	0.188	-0.726	0	-2	-2
Norway	19.353	3.784	+/+	5.114	0.709	1	1	2
Oman	36.368	98.875	+/+	0.368	-0.434	1	-2	-1
Portugal	8.334	0.251	+/+	33.202	1.521	1	2	3
Qatar	103.128	98.848	+/+	1.043	0.018	1	0	1
Reunion	15.461	1.033	+/+	14.961	1.175	1	2	3
Russian Federation	7.675	3.743	+/+	2.051	0.312	1	1	2
Rwanda	10.983	3.002	+/+	3.658	0.563	1	1	2
Saudi Arabia	957.991	1158.326	+/+	0.827	-0.082	1	-1	0
Singapore	1.432	2.626	+/+	0.545	-0.263	0	-1	-1
Slovenia	10.899	21.741	+/+	0.501	-0.300	1	-1	0
Solomon Islands	0.200	0.211	+/+	0.948	-0.023	0	-1	-1
Spain	4.584	3.677	+/+	1.247	0.096	1	0	1
Sweden	29.795	50.471	+/+	0.590	-0.229	1	-1	0
Switzerland	4.409	4.150	+/+	1.063	0.026	1	0	1
Togo	0.649	0.861	+/+	0.754	-0.123	0	-1	-1
Ukraine	2.092	1.655	+/+	1.264	0.102	1	0	1
United Arab Emirates	3220.696	473.981	+/+	6.795	0.832	1	1	2
United Kingdom	9.894	3.165	+/+	3.126	0.495	1	1	2

United States	16.283	5.617	+/+	2.899	0.462	1	1	2
Venezuela, RB	0.877	1.733	+/+	0.506	-0.296	0	-1	-1

Country	HbS slope	MIG slope	Group	Ratio (R)/Difference (D)	LogR/LogD	HbS Index	DIV Index	Index of Change
Armenia	57.412	-5.674	+/-	63.086	1.800	1	2	3
Bangladesh	0.053	-22.918	+/-	22.971	1.361	0	2	2
Bolivia	4.277	-5.994	+/-	10.271	1.012	1	1	2
Chile	0.765	-10.970	+/-	11.734	1.069	0	1	1
China	11.087	-0.855	+/-	11.942	1.077	1	1	2
Ecuador	6.407	-39.363	+/-	45.770	1.661	1	2	3
Eritrea	0.760	-1.265	+/-	2.025	0.307	0	0	0
Estonia	3.474	-0.163	+/-	3.636	0.561	1	0	1
Iceland	16.438	-0.105	+/-	16.543	1.219	1	1	2
Ireland	58.143	-0.619	+/-	58.762	1.769	1	2	3
Korea, Dem. Rep.	24.889	-8.548	+/-	33.437	1.524	1	2	3
Korea, Rep.	4.229	-2.382	+/-	6.611	0.820	1	1	2
Kyrgyz Republic	0.642	-4.260	+/-	4.902	0.690	0	0	0
Latvia	1.357	-0.386	+/-	1.743	0.241	0	0	0
Mauritania	0.939	-0.431	+/-	1.370	0.137	0	0	0
Mauritius	5.443	-99.925	+/-	105.367	2.023	1	2	3
New Zealand	1.194	-1.335	+/-	2.529	0.403	0	0	0
Niger	0.625	-1.717	+/-	2.342	0.370	0	0	0
Paraguay	14.780	-1.050	+/-	15.830	1.199	1	1	2
Philippines	30.805	-116.262	+/-	147.067	2.168	1	2	3
Poland	1.948	-0.635	+/-	2.584	0.412	0	0	0
Puerto Rico	1.460	-2.367	+/-	3.827	0.583	0	0	0
Samoa	4.667	-31.754	+/-	36.421	1.561	1	2	3
Serbia and Montenegro	7.606	-5.124	+/-	12.730	1.105	1	1	2
Slovak Republic	0.818	-0.086	+/-	0.904	-0.044	0	0	0
Somalia	5.499	-5.960	+/-	11.458	1.059	1	1	2
South Africa	1.038	-1.022	+/-	2.060	0.314	0	0	0
Swaziland	8.405	-0.284	+/-	8.689	0.939	1	1	2
Thailand	30.996	-2.774	+/-	33.770	1.529	1	2	3
Timor-Leste	0.667	-4.350	+/-	5.017	0.700	0	1	1
Tonga	51.000	-49.984	+/-	100.984	2.004	1	2	3
Turkmenistan	4.244	-12.657	+/-	16.901	1.228	1	1	2
Uzbekistan	26.075	-3.550	+/-	29.625	1.472	1	2	3
Vietnam	14.882	-13.844	+/-	28.726	1.458	1	2	3
Yemen, Rep.	0.161	-10.792	+/-	10.954	1.040	0	1	1

Country	HbS slope	MIG slope	Group	Ratio (R)/Difference (D)	LogR/LogD	HbS Index	DIV Index	Index of Change
Afghanistan	-3.366	-81.141	-/-	0.041	-1.382	-1	2	1
Albania	-262.601	-73.950	-/-	3.551	0.550	-1	-2	-3
Algeria	-3.943	-3.769	-/-	1.046	0.020	-1	-1	-2
Angola	-13.805	-38.612	-/-	0.358	-0.447	-1	1	0
Argentina	-1.700	-1.534	-/-	1.108	0.045	0	-1	-1
Azerbaijan	-12.873	-107.913	-/-	0.119	-0.923	-1	1	0
Barbados	-7.311	-7.396	-/-	0.988	-0.005	-1	-1	-2
Belize	-22.251	-33.672	-/-	0.661	-0.180	-1	0	-1
Benin	-6.453	-6.436	-/-	1.003	0.001	-1	-1	-2
Bosnia and Herzegovina	-11.855	-12.193	-/-	0.972	-0.012	-1	0	-1
Brazil	-5.330	-2.916	-/-	1.828	0.262	-1	-1	-2
Bulgaria	-1.321	-1.255	-/-	1.052	0.022	0	-1	-1
Burkina Faso	-1.332	-1.959	-/-	0.680	-0.167	0	0	0
Burundi	-13.872	-3.072	-/-	4.516	0.655	-1	-2	-3
Cambodia	-1.621	-3.652	-/-	0.444	-0.353	0	0	0
Cameroon	-2.214	-1.514	-/-	1.463	0.165	-1	-1	-2
Cape Verde	-8.773	-7.615	-/-	1.152	0.062	-1	-1	-2
Central African Republic	-0.555	-0.573	-/-	0.968	-0.014	0	0	0
Chad	-2.325	-9.887	-/-	0.235	-0.629	-1	1	0
Colombia	-28.584	-25.546	-/-	1.119	0.049	-1	-1	-2
Congo, Dem. Rep.	-4.742	-3.895	-/-	1.218	0.086	-1	-1	-2
Cuba	-36.627	-13801.743	-/-	0.003	-2.576	-1	2	1
Cyprus	-5.098	-2.776	-/-	1.836	0.264	-1	-1	-2
Dominican Republic	-4.900	-17.390	-/-	0.282	-0.550	-1	1	0
Egypt, Arab Rep.	-34.550	-470.477	-/-	0.073	-1.134	-1	1	0
El Salvador	-490.440	-73.415	-/-	6.680	0.825	-1	-2	-3
Equatorial Guinea	-66.040	-11.102	-/-	5.949	0.774	-1	-2	-3

Ethiopia	-0.032	-1.475	-/-	0.022	-1.658	0	2	2
Fiji	-1.504	-26.774	-/-	0.056	-1.250	0	1	1
Georgia	-9.563	-10.224	-/-	0.935	-0.029	-1	0	-1
Ghana	-4.546	-5.421	-/-	0.839	-0.076	-1	0	-1
Grenada	-5.560	-5.693	-/-	0.977	-0.010	-1	-1	-2
Guadeloupe	-6.122	-4.790	-/-	1.278	0.107	-1	-1	-2
Guatemala	-20.855	-133.211	-/-	0.157	-0.805	-1	1	0
Guinea	-1.561	-0.490	-/-	3.183	0.503	0	-2	-2
Guinea-Bissau	-6.445	-2.611	-/-	2.468	0.392	-1	-2	-3
Guyana	-30.397	-45.587	-/-	0.667	-0.176	-1	0	-1
Haiti	-11.539	-12.166	-/-	0.948	-0.023	-1	0	-1
Honduras	-22.539	-58.116	-/-	0.388	-0.411	-1	1	0
Hungary	-1.919	-0.670	-/-	2.865	0.457	0	-2	-2
India	-0.282	-27.032	-/-	0.010	-1.982	0	2	2
Indonesia	-2.264	-5.199	-/-	0.436	-0.361	-1	0	-1
Iraq	-19.980	-20.232	-/-	0.988	-0.005	-1	-1	-2
Jamaica	-9.086	-9.531	-/-	0.953	-0.021	-1	0	-1
Jordan	-15.050	-13.702	-/-	1.098	0.041	-1	-1	-2
Kazakhstan	-0.410	-3.860	-/-	0.106	-0.973	0	1	1
Lao PDR	-7.667	-288.605	-/-	0.027	-1.576	-1	2	1
Lebanon	-9.667	-5.454	-/-	1.772	0.249	-1	-1	-2
Lesotho	-0.251	-0.383	-/-	0.656	-0.183	0	0	0
Liberia	-4.785	-34.515	-/-	0.139	-0.858	-1	1	0
Lithuania	-1.024	-0.958	-/-	1.070	0.029	0	-1	-1
Macedonia, FYR	-2.531	-2.554	-/-	0.991	-0.004	-1	-1	-2
Madagascar	-9.931	-3.277	-/-	3.030	0.482	-1	-2	-3
Mali	-28.913	-13.764	-/-	2.101	0.322	-1	-1	-2
Martinique	-9.573	-8.113	-/-	1.180	0.072	-1	-1	-2
Mexico	-65.948	-50.099	-/-	1.316	0.119	-1	-1	-2
Micronesia, Fed. Sts.	-2.263	-8.771	-/-	0.258	-0.588	-1	1	0
Moldova	-1.943	-1.507	-/-	1.289	0.110	0	-1	-1
Morocco	-8.638	-12.177	-/-	0.709	-0.149	-1	0	-1
Myanmar	-1.914	-5.898	-/-	0.325	-0.489	0	1	1
Netherlands Antilles	-23.457	-46.544	-/-	0.504	-0.298	-1	0	-1
Nicaragua	-37.559	-35.741	-/-	1.051	0.022	-1	-1	-2
Panama	-33.693	-8.900	-/-	3.786	0.578	-1	-2	-3
Papua New Guinea	-0.788	-7.002	-/-	0.112	-0.949	0	1	1
Peru	-0.497	-150.320	-/-	0.003	-2.481	0	2	2
Romania	-19.334	-11.834	-/-	1.634	0.213	-1	-1	-2
Sao Tome and Principe	-1.371	-1.987	-/-	0.690	-0.161	0	0	0
Senegal	-3.730	-5.830	-/-	0.640	-0.194	-1	0	-1
Sierra Leone	-9.254	-6.817	-/-	1.357	0.133	-1	-1	-2
Sri Lanka	-1.984	-4.045	-/-	0.490	-0.309	0	0	0
St. Lucia	-9.218	-9.564	-/-	0.964	-0.016	-1	0	-1
St. Vincent and the Grenadines	-5.961	-6.130	-/-	0.972	-0.012	-1	0	-1
Sudan	-1.623	-5.213	-/-	0.311	-0.507	0	1	1
Suriname	-29.786	-255.815	-/-	0.116	-0.934	-1	1	0
Syrian Arab Republic	-11.545	-14.943	-/-	0.773	-0.112	-1	0	-1
Tajikistan	-2.680	-4.992	-/-	0.537	-0.270	-1	0	-1
Tanzania	-4.546	-2.427	-/-	1.873	0.272	-1	-1	-2
Trinidad and Tobago	-45.391	-373.774	-/-	0.121	-0.916	-1	1	0
Tunisia	-14.566	-17.356	-/-	0.839	-0.076	-1	0	-1
Turkey	-4.852	-9.706	-/-	0.500	-0.301	-1	0	-1
Uganda	-3.707	-3.034	-/-	1.222	0.087	-1	-1	-2
Uruguay	-1.912	-6.113	-/-	0.313	-0.505	0	1	1
Vanuatu	-2.143	-10.529	-/-	0.204	-0.691	-1	1	0
Zambia	-6.906	-3.040	-/-	2.272	0.356	-1	-1	-2
Zimbabwe	-1.192	-2.480	-/-	0.481	-0.318	0	0	0

Country	HbS slope	MIG slope	Group	Ratio (R)/Difference (D)	LogR/LogD	HbS Index	DIV Index	Index of Change
Belarus	-0.448	0.915	-/+	1.363	0.135	0	-1	-1
Iran, Islamic Rep.	-10.156	2.896	-/+	13.052	1.116	-1	-2	-3
Israel	-39.513	1.176	-/+	40.689	1.609	-1	-2	-3
Maldives	-1.725	0.032	-/+	1.757	0.245	0	-1	-1
Pakistan	-1.434	2.354	-/+	3.788	0.578	0	-1	-1

Supplementary Table 2: Net number of migrants and estimated net number of migrants with HbS (median, low and high estimates) per country based on the World Bank Global Bilateral Migration Database and HbS estimates published by Piel et al (2013).

	Net number of migrants					Net number of migrants with HbS														
						Median estimate					Lower-bound estimate					Higher-bound estimate				
**Country**	**1960**	**1970**	**1980**	**1990**	**2000**	**AS1960med**	**AS1970med**	**AS1980med**	**AS1990med**	**AS2000med**	**AS1960low**	**AS1970low**	**AS1980low**	**AS1990low**	**AS2000low**	**AS1960high**	**AS1970high**	**AS1980high**	**AS1990high**	**AS2000high**
Afghanistan	-37,601	-78,105	-315,000	-996,648	-1,103,820	1,055	1,267	264	-178	2	889	1,078	257	51	225	1,270	1,480	113	-1,042	-915
Albania	-26,782	-5,263	10,184	-140,238	-949,565	208	914	1,461	-2,659	-25,316	400	887	1,267	-1,345	-15,879	-20	1,037	1,845	-4,582	-39,726
Algeria	-428,683	-1,532,834	-1,477,745	-1,469,474	-1,268,206	-5,883	-21,632	-20,867	-20,759	-17,917	-5,009	-16,396	-15,775	-15,651	-13,500	-6,392	-29,514	-28,594	-28,569	-24,686
Angola	23,103	-85,483	-189,689	-248,285	-341,523	-13,013	-27,532	-47,710	-61,828	-85,687	-10,658	-20,889	-35,672	-46,193	-64,053	-14,511	-35,175	-62,319	-80,846	-111,953
Argentina	2,444,244	1,975,928	1,577,327	1,198,413	957,917	27,552	21,055	15,724	12,036	8,640	17,369	13,228	9,824	7,494	5,318	46,372	35,710	26,977	20,755	15,235
Armenia	-166,079	-54,085	-34,134	-203,223	-562,693	-34	-9	-1	-53	964	-9	-2	0	-15	746	-146	-42	-17	-222	1,037
Aruba	5,818	5,992	2,371	4,923	19,068	213	221	108	207	581	125	130	53	119	364	376	381	209	370	971
Australia	1,533,053	2,236,139	2,671,213	3,256,766	3,552,684	26,017	40,590	46,502	50,576	52,629	18,156	28,486	32,836	35,759	37,500	38,975	60,559	68,641	74,575	77,026
Austria	147,563	46,899	99,980	289,387	463,643	6,188	3,772	9,102	4,826	6,962	4,273	2,722	5,957	3,606	5,146	8,222	4,531	13,474	6,342	9,622
Azerbaijan	-26,972	-147,996	-348,782	-618,690	-1,246,938	-79	-123	-196	-296	-501	-14	-26	-43	-67	-121	-329	-525	-782	-1,184	-1,964
Bahrain	23,763	32,690	84,265	146,418	186,087	231	186	746	1,564	835	204	193	645	1,329	959	302	211	1,005	2,058	932
Bangladesh	608,177	491,207	-4,242,780	-4,186,918	-4,021,878	28,530	31,192	24,029	27,345	31,203	24,471	26,915	24,840	27,696	30,929	33,705	36,466	17,796	21,666	26,419
Barbados	-20,654	-13,171	-50,939	-71,726	-67,760	-979	-554	-2,322	-3,367	-3,151	-297	-73	-689	-1,080	-961	-2,698	-1,861	-6,424	-9,038	-8,644
Belarus	-882,905	-1,154,590	-1,022,926	-898,258	-606,999	230	124	159	197	142	87	60	71	83	58	556	141	240	335	247
Belgium	-541,331	210,631	482,030	480,749	538,166	-9,824	3,943	9,016	9,856	11,905	-8,543	2,016	5,828	6,605	8,274	-10,856	7,835	15,077	16,009	18,387
Belize	1,776	-19,973	-9,548	-41,297	-17,463	-569	-3,867	-2,600	-5,730	-5,968	-408	-2,711	-1,831	-3,990	-4,283	-769	-5,458	-3,635	-8,187	-8,222
Benin	-63,649	-102,504	-330,876	-192,079	-223,688	-19,720	-30,904	-92,912	-57,332	-70,131	-16,745	-26,232	-78,732	-48,655	-59,576	-22,552	-35,411	-107,038	-65,744	-80,137
Bhutan	9,396	11,862	6,985	9,137	20,447	411	554	735	991	1,351	351	475	636	860	1,165	486	652	852	1,151	1,582
Bolivia	-78,248	-87,720	-108,790	-167,248	-273,010	365	485	573	706	1,035	265	353	428	530	773	505	666	777	947	1,377
Bosnia and Herzegovina	-206,271	-343,779	-289,777	-771,783	-1,249,798	-1,005	-1,707	-1,396	-3,513	-6,059	-300	-530	-407	-905	-1,791	-2,997	-5,021	-4,196	-11,001	-18,128
Botswana	-59,803	-46,833	-76,875	-47,485	13,845	-101	-48	-457	755	3,157	46	64	-213	762	2,543	-487	-340	-1,004	536	3,867
Brazil	1,252,498	1,001,820	785,175	318,466	-231,849	16,092	11,018	6,837	-3,445	-19,565	9,819	6,256	3,203	-4,210	-16,574	27,489	19,973	13,877	-956	-22,519
Brunei Darussalam	19,631	27,493	42,873	-13,906	52,994	32	65	125	184	263	25	53	93	142	204	44	86	175	250	359
Bulgaria	-475,954	-446,718	-382,081	-630,717	-682,703	-6,272	-5,895	-5,051	-8,297	-9,213	-2,728	-2,556	-2,181	-3,614	-3,821	-14,148	-13,313	-11,437	-18,678	-21,273
Burkina Faso	-396,672	-704,888	-734,017	-637,533	-818,842	-40,237	-72,064	-73,109	-54,742	-75,696	-35,888	-64,081	-65,663	-51,219	-72,562	-44,947	-80,781	-80,981	-57,755	-76,881
Burundi	-67,661	-138,618	-220,062	-159,059	-161,365	-1,425	-7,677	-14,590	-9,621	-10,337	701	-4,205	-9,516	-5,985	-6,707	-4,904	-12,971	-22,056	-15,017	-15,664
Cambodia	363,554	300,388	-90,221	-208,984	-45,661	145	118	-1	9	82	97	77	0	8	58	237	193	-5	3	122
Cameroon	115,376	132,970	18,865	101,567	43,762	24,245	27,949	3,813	15,066	3,845	21,215	24,465	3,149	12,691	2,850	27,837	32,073	4,808	18,095	5,286
Canada	1,633,060	2,120,129	2,575,073	3,335,136	4,211,715	27,964	41,704	55,916	75,705	101,240	20,563	30,860	41,575	55,182	75,080	39,993	59,100	78,763	109,013	143,192
Cape Verde	-34,122	-31,781	-55,778	-75,763	-142,045	-1,349	-1,189	-2,356	-3,092	-6,315	-893	-771	-1,605	-2,388	-4,732	-1,956	-1,760	-3,365	-3,885	-8,222
Central African Republic	19,445	-13,673	13,614	25,024	-5,475	3,447	-1,551	2,748	4,424	-497	3,424	-500	3,144	4,396	77	3,301	-3,071	2,019	4,225	-1,345
Chad	-14,880	-29,896	-56,643	-91,271	-57,750	3,422	2,480	441	-2,301	1,834	3,780	3,197	1,817	-80	3,238	2,806	1,362	-1,533	-5,400	-222
Chile	-69,361	-144,767	-321,722	-380,779	-331,784	1,147	863	786	1,050	1,492	751	557	526	717	1,019	1,848	1,403	1,249	1,608	2,297
China	-4,557,645	-2,558,853	-4,120,431	-4,370,875	-5,601,102	161	84	-71	91	1,050	178	105	-5	125	897	42	16	-252	-46	1,157
Colombia	-127,759	-257,132	-630,881	-925,957	-1,425,231	-1,720	-3,564	-9,187	-13,577	-21,296	-804	-1,754	-4,873	-7,285	-11,698	-3,407	-6,877	-16,978	-24,947	-38,521
Comoros	-48,651	-35,508	-5,444	-25,739	-41,043	-1,600	-981	900	60	-496	-942	-497	970	386	36	-2,842	-1,908	633	-653	-1,598
Congo, Dem. Rep.	677,578	665,251	249,148	26,204	-322,314	-53,902	-19,368	-47,820	-73,279	-154,748	-49,674	-20,962	-44,702	-66,535	-138,467	-56,854	-15,141	-49,643	-79,381	-171,160
Congo, Rep.	-30,380	-18,001	32,372	72,512	-39,955	-9,302	-7,442	6,010	14,637	-10,520	-7,276	-5,632	5,713	13,237	-8,197	-11,895	-9,800	6,109	15,925	-13,497
Costa Rica	5,068	-16,467	1,375	65,008	189,879	488	-354	395	3,285	8,827	269	-151	195	1,390	3,710	899	-798	743	7,236	19,561
Cote d'Ivoire	642,276	1,061,343	1,347,561	1,387,538	1,657,697	84,459	138,511	176,061	187,227	219,622	72,370	118,021	150,128	160,328	190,744	97,551	161,134	204,597	216,171	249,663
Croatia	-74,338	-190,632	-148,760	96,956	-1,732	155	-331	222	532	168	288	135	505	287	154	-468	-1,899	-1,020	1,023	26
Cuba	179	-479,129	-730,435	-872,843	-1,038,220	-3,726	-31,583	-44,680	-52,208	-61,650	-1,501	-17,564	-25,387	-29,863	-35,377	-7,957	-56,506	-78,727	-91,534	-107,860
Cyprus	-64,978	-82,117	-115,633	-163,694	-114,385	-460	-680	-1,134	-1,775	-1,085	-62	-163	-392	-722	-270	-1,282	-1,730	-2,603	-3,837	-2,725
Czech Republic	-1,137,777	-1,114,861	-979,458	-186,032	-426,075	-1,731	-1,699	-1,493	-277	-189	-489	-477	-419	42	240	-6,240	-6,125	-5,401	-1,703	-2,285
Denmark	-132,867	-89,836	-91,816	-1,650	128,822	563	537	1,280	2,890	5,053	457	404	998	2,116	3,604	443	519	1,447	3,843	7,243
Djibouti	10,144	19,736	38,016	65,550	82,415	297	536	1,091	1,717	2,204	238	433	874	1,375	1,768	385	703	1,415	2,238	2,866
Dominican Republic	86,082	72,494	-23,425	-206,905	-522,704	8,246	9,254	6,861	1,628	-8,144	6,690	8,070	7,645	6,106	2,639	9,566	9,209	2,273	-11,227	-34,804
Ecuador	-28,305	-37,908	-79,187	-152,658	-528,015	297	568	868	945	1,060	201	381	590	666	835	426	824	1,237	1,250	915
Egypt, Arab Rep.	12,490	-229,900	-885,530	-1,788,491	-2,146,345	4,143	-2,189	-18,160	-39,524	-48,760	4,223	715	-7,503	-18,137	-22,698	3,223	-8,549	-39,528	-81,773	-100,006
El Salvador	-30,608	-40,816	-129,840	-541,849	-903,635	125	169	-2,593	-13,900	-23,493	-49	-42	-1,725	-8,743	-14,763	554	656	-3,829	-21,866	-37,002
Equatorial Guinea	15,963	-4,377	-19,625	-32,696	-58,485	-646	-4,151	-6,855	-10,535	-18,785	-540	-3,298	-5,393	-8,338	-14,881	-708	-5,019	-8,372	-12,861	-22,931
Eritrea	-229,142	-262,990	-280,298	-313,694	-348,770	-725	-624	-561	-257	-633	-175	-11	70	394	140	-2,070	-2,135	-2,160	-1,991	-2,615
Estonia	87,506	95,485	176,941	214,983	20,644	19	29	36	47	43	7	10	15	17	21	55	75	101	135	91
Ethiopia	378,549	342,576	326,530	254,833	143,251	10,089	9,501	9,819	9,872	9,740	7,901	7,453	7,718	7,808	7,776	13,663	12,861	13,269	13,270	12,997
Fiji	11,641	3,151	-14,488	-61,129	-112,055	617	423	258	217	256	507	348	213	179	210	773	528	323	274	317
Finland	-248,598	-348,753	-305,597	-259,951	-242,238	137	157	221	390	1,017	104	118	154	286	746	182	219	329	553	1,474
France	2,300,687	4,028,874	4,451,846	4,486,562	4,501,951	17,053	49,700	85,935	95,483	52,100	12,651	35,424	64,226	73,276	37,977	26,225	75,551	122,108	131,660	77,615
French Guiana	6,048	13,691	19,010	45,607	71,802	356	702	1,018	2,339	3,640	233	474	675	1,557	2,425	564	1,054	1,558	3,603	5,593
French Polynesia	1,351	7,305	6,820	20,197	16,803	48	166	404	505	612	34	115	283	356	428	71	247	598	754	897
Gabon	17,260	32,909	57,979	113,338	178,874	924	5,389	9,537	20,803	34,228	753	4,381	7,722	16,985	28,069	1,228	6,628	11,814	25,385	41,507
Gambia, The	19,722	33,581	55,630	94,742	134,893	2,814	3,124	8,352	14,567	20,778	2,110	2,258	6,465	11,519	16,397	3,614	4,244	10,513	18,018	25,710
Georgia	275,645	-85,205	-243,489	-510,860	-920,608	48	-6	-37	-77	-146	18	2	-5	-18	-35	144	-73	-175	-337	-589
Germany	1,660,498	3,738,988	5,646,085	5,745,015	7,525,986	-8,027	21,728	45,252	37,359	85,101	-2,779	18,336	36,142	30,591	67,439	-14,913	30,761	64,762	52,876	116,744

	Net number of migrants					Net number of migrants with HbS														
						Median estimate					Lower-bound estimate					Higher-bound estimate				
Country	1960	1970	1980	1990	2000	AS1960med	AS1970med	AS1980med	AS1990med	AS2000med	AS1960low	AS1970low	AS1980low	AS1990low	AS2000low	AS1960high	AS1970high	AS1980high	AS1990high	AS2000high
Ghana	320,091	217,083	-317,240	-267,179	-305,337	73,200	38,401	-50,256	-44,059	-51,936	62,931	31,861	-46,239	-41,356	-49,286	84,852	45,963	-54,433	-46,466	-53,923
Greece	-913,343	-1,097,746	-1,011,557	-645,758	-318,931	-48,345	-58,787	-57,478	-46,924	-37,647	-37,077	-45,053	-44,164	-36,444	-30,555	-66,948	-81,408	-79,421	-64,193	-49,567
Grenada	-16,738	-16,408	-33,329	-41,016	-52,075	-1,234	-1,201	-2,387	-2,956	-3,787	-526	-515	-1,059	-1,285	-1,621	-2,598	-2,529	-4,962	-6,174	-7,982
Guadeloupe	-43,582	-10,126	-81,944	32,490	-169,259	-2,729	-1,027	-5,523	755	-11,973	-1,957	-745	-3,973	526	-8,613	-3,806	-1,412	-7,680	1,148	-16,610
Guam	13,525	13,113	16,461	54,951	17,279	493	94	514	646	458	385	67	406	506	358	638	136	660	836	599
Guatemala	9,835	-13,033	-57,742	-265,417	-519,038	1,422	391	-666	-5,175	-10,623	819	306	-295	-2,784	-5,792	2,459	482	-1,390	-9,516	-19,336
Guinea	-185,480	-230,408	-307,736	-334,965	-178,686	-53,609	-67,055	-89,992	-102,649	-77,656	-42,962	-53,751	-72,153	-82,935	-64,604	-64,382	-80,508	-108,033	-122,346	-89,808
Guinea-Bissau	-30,981	-36,975	-34,392	-56,261	-61,784	-1,111	-1,507	-1,237	-3,315	-3,787	-600	-877	-671	-2,199	-2,542	-1,948	-2,519	-2,168	-4,973	-5,611
Guyana	-19,495	-8,558	-104,670	-241,740	-347,265	-1,156	-521	-4,215	-9,790	-14,091	-442	-148	-1,725	-4,115	-5,917	-2,831	-1,449	-9,933	-22,744	-32,754
Haiti	-127,452	-200,111	-289,508	-498,831	-753,358	-13,143	-19,972	-28,780	-49,306	-74,303	-10,013	-15,095	-21,741	-37,200	-56,037	-17,094	-26,272	-37,898	-65,035	-98,066
Honduras	12,374	-52,514	-61,317	-121,303	-312,797	-2,841	-6,280	-7,036	-13,414	-31,291	-1,481	-3,444	-3,888	-7,442	-17,212	-5,206	-11,144	-12,428	-23,552	-55,483
Hong Kong SAR, China	1,575,659	1,630,219	1,808,961	1,782,248	1,941,429	539	957	694	1,103	4,714	376	726	488	805	3,725	817	1,311	1,052	1,587	6,219
Hungary	-105,510	-109,884	-102,765	-104,679	-143,438	7,292	2,189	1,443	1,059	862	5,442	1,664	1,106	832	710	9,838	2,675	1,663	1,128	738
Iceland	-12,942	-10,512	-13,405	-14,721	-11,519	16	27	38	68	127	11	19	26	49	88	28	46	62	107	191
India	328,571	862,845	1,263,332	-799,115	-3,281,399	-361,255	-314,898	-306,271	-339,016	-400,079	-324,427	-283,998	-272,465	-298,786	-349,621	-389,544	-336,787	-337,275	-380,660	-456,604
Indonesia	1,547,401	930,701	246,126	-620,642	-1,699,141	3,819	2,397	1,191	187	600	3,174	1,990	1,021	250	547	4,729	2,944	1,371	-27	450
Iran, Islamic Rep.	-123,170	-146,908	-204,279	85,496	-61,014	-3,524	-4,724	-10,386	-12,580	-17,490	-2,164	-2,913	-6,435	-7,909	-11,103	-5,795	-7,752	-16,846	-19,896	-27,241
Iraq	-109,216	-192,938	-371,333	-595,757	-1,012,615	-2,192	-3,444	-6,763	-10,740	-20,442	-818	-1,549	-3,306	-5,355	-10,498	-5,482	-7,869	-14,572	-22,850	-42,126
Ireland	-466,299	-992,967	-688,546	-736,016	-739,076	259	111	2,295	2,314	6,687	665	1,057	2,495	2,555	6,291	-1,812	-4,372	-494	-701	4,130
Israel	968,725	1,124,618	1,051,231	1,118,181	1,541,616	967	-4	1,550	-6,887	-14,696	-648	-1,598	-90	-6,017	-11,718	6,663	6,378	7,753	-4,341	-14,484
Italy	-4,044,030	-4,447,919	-3,401,039	-2,360,000	-1,018,579	-37,871	-38,303	-22,259	-2,708	20,206	-22,511	-21,854	-10,602	3,754	19,989	-67,279	-70,393	-45,926	-17,439	17,294
Jamaica	-188,971	-246,716	-404,536	-612,070	-906,835	-14,096	-18,519	-29,552	-44,083	-65,350	-11,193	-14,671	-23,332	-34,711	-51,451	-17,777	-23,442	-37,592	-56,275	-83,437
Japan	349,622	300,790	198,088	352,462	811,975	347	711	1,054	3,566	11,689	267	557	819	2,764	9,122	455	935	1,387	4,731	15,344
Jordan	-139,578	-331,217	-561,721	-826,050	-848,390	-4,700	-11,282	-19,378	-29,084	-31,166	-3,227	-7,786	-13,442	-20,347	-21,841	-6,976	-16,617	-28,330	-42,054	-44,877
Kazakhstan	835,753	1,266,773	1,256,638	806,810	-547,115	268	359	368	359	213	89	121	124	123	75	830	1,129	1,150	1,137	670
Kenya	-244,482	-231,488	-163,851	-155,426	166,194	-14,907	-16,054	-9,924	-8,041	41,017	-13,535	-14,890	-9,480	-7,820	32,498	-16,465	-17,245	-10,324	-8,211	50,934
Korea, Dem. Rep.	-137,000	-151,838	-527,686	-580,663	-508,119	9	17	19	43	108	7	12	12	32	83	14	22	23	54	139
Korea, Rep.	-701,269	-610,937	-563,706	-1,008,875	-1,337,567	153	68	119	135	443	118	49	88	97	333	201	92	169	184	611
Kuwait	23,531	367,845	741,835	1,221,814	1,056,230	-1,441	9,472	19,580	26,275	18,995	-223	7,666	15,470	21,841	17,862	-4,062	11,258	24,456	30,342	16,809
Kyrgyz Republic	381,518	379,738	369,464	112,482	-297,553	53	55	54	45	75	17	17	19	17	56	160	158	152	110	76
Lao PDR	-3,054	-7,234	-111,611	-266,747	-313,997	3	3	2	-4	-5	3	3	3	2	1	6	5	-7	-27	-36
Latvia	233,238	237,150	325,231	423,231	95,216	70	87	95	118	102	24	29	33	41	51	225	258	299	367	207
Lebanon	-127,077	-184,221	-430,951	-389,666	-370,914	-12,789	-18,142	-40,793	-52,147	-57,604	-8,769	-12,452	-27,967	-35,768	-39,463	-18,099	-25,693	-57,851	-73,404	-80,978
Lesotho	-200,107	-189,361	-213,264	-322,564	-171,776	-295	-293	-359	-501	-228	-60	-70	-94	-109	-26	-983	-942	-1,097	-1,615	-821
Liberia	12,887	27,899	-11,984	-12,660	-189,234	3,692	8,133	10,125	10,060	-6,104	2,902	6,452	7,886	7,837	-5,456	4,511	9,875	12,216	12,145	-7,569
Libya	-6,951	39,380	216,298	350,466	390,843	-1,344	46	4,809	8,380	8,863	-1,081	-403	2,516	4,680	4,731	-1,548	1,260	9,327	15,415	16,940
Lithuania	-149,719	-193,780	-37,560	25,113	-330,844	123	132	149	166	43	42	46	50	56	42	365	374	455	511	-24
Luxembourg	13,065	30,377	48,981	81,240	116,370	335	599	1,059	1,766	2,378	250	421	733	1,252	1,674	493	920	1,619	2,630	3,547
Macao SAR, China	60,148	-1,809,160	99,686	24,098	143,340	39	10	59	115	81	29	11	41	75	56	60	-5	88	178	126
Macedonia, FYR	-100,608	-133,921	-448,597	-128,510	-231,813	-2,080	-2,747	-9,069	-2,670	-4,751	-1,075	-1,420	-4,745	-1,075	-2,118	-4,004	-5,270	-17,352	-5,772	-9,843
Madagascar	116,475	55,604	-25,303	-42,858	-25,146	2,832	329	-7,135	-8,990	-6,571	1,834	-79	-6,418	-8,027	-5,893	4,588	1,156	-7,826	-9,920	-7,193
Malawi	1,795	-16,939	23,218	7,901	43,885	-96	-1,293	121	-837	1,496	1,937	1,075	1,868	1,176	2,761	-4,773	-6,479	-3,835	-5,201	-1,644
Malaysia	-166,172	421,552	264,641	356,510	343,929	537	6,915	4,655	2,062	4,105	469	5,879	3,934	1,644	3,294	612	8,288	5,625	2,697	5,336
Maldives	1,642	1,766	1,332	559	2,272	40	47	14	14	22	33	39	11	8	16	50	60	24	21	33
Mali	-94,487	-235,010	-493,795	-488,004	-618,263	-4,744	-19,839	-47,560	-47,042	-59,724	-3,650	-15,584	-37,493	-37,075	-46,971	-5,832	-24,736	-59,439	-58,786	-74,979
Malta	-79,514	-93,721	-98,519	-103,864	-62,388	-2,355	-2,875	-2,971	-3,122	-1,941	-1,153	-1,370	-1,438	-1,513	-921	-4,825	-5,982	-6,122	-6,427	-4,041
Martinique	-20,902	-8,181	-92,111	13,141	-116,357	-1,649	-1,036	-7,039	-450	-9,835	-1,200	-760	-5,130	-343	-7,173	-2,253	-1,381	-9,579	-547	-13,354
Mauritania	-31,450	-23,414	-34,246	-32,044	-33,909	-2,692	-1,738	-2,443	-1,522	-1,536	-1,346	-568	-670	461	598	-5,015	-3,852	-5,684	-5,367	-5,703
Mauritius	-1,794	-7,669	-57,600	-94,380	-48,071	174	176	217	151	660	137	142	192	152	571	210	199	143	-19	629
Mayotte	1,228	2,658	5,674	13,711	39,023	57	124	269	645	1,704	40	87	193	482	1,201	82	183	398	904	2,546
Mexico	-415,823	-839,784	-2,310,001	-4,400,061	-9,051,811	-4,328	-10,214	-30,374	-58,869	-122,711	-3,705	-8,233	-23,844	-46,133	-95,623	-4,915	-12,738	-39,256	-76,287	-160,190
Micronesia, Fed. Sts.	4,810	2,817	-6,927	-4,509	-12,621	80	73	47	18	17	60	52	32	14	13	122	107	65	24	20
Moldova	-87,727	-32,375	48,093	-10,413	-164,790	-87	-85	-66	-116	-156	-11	-10	-3	-16	-27	-394	-399	-342	-535	-687
Mongolia	3,273	2,562	3,451	4,690	3,337	6	7	9	10	14	4	6	7	8	9	7	10	13	15	19
Morocco	-282,260	-705,812	-1,171,998	-1,586,799	-1,560,375	3,513	-2,541	-6,054	-8,674	-8,594	3,664	-251	-1,947	-3,033	-3,024	821	-9,302	-17,299	-23,903	-23,560
Mozambique	-640,876	-629,795	-447,951	-448,768	-390,304	-34,123	-33,413	-24,179	-26,128	-28,381	-26,365	-25,827	-18,736	-20,271	-21,957	-43,944	-42,998	-30,970	-33,313	-36,110
Myanmar	154,603	76,835	-59,266	-68,678	-228,570	4,929	4,667	3,090	2,000	1,545	4,197	3,983	2,655	1,724	1,356	5,881	5,543	3,625	2,331	1,728
Namibia	6,204	6,498	3,537	4,886	43,664	3,360	5,322	8,403	13,459	13,621	2,586	4,106	6,496	10,401	10,447	4,267	6,735	10,597	16,968	17,389
Nepal	-187,132	-262,796	-395,764	-174,890	-179,689	12,822	12,529	8,324	16,385	22,907	11,710	11,555	7,999	14,844	20,657	13,405	12,822	7,731	17,400	24,578
Netherlands	-198,625	-436,083	-235,710	454,544	810,161	-11,771	-11,782	-7,576	6,597	19,168	-8,501	-8,617	-6,115	2,492	11,713	-15,850	-15,384	-7,688	17,558	35,560
Netherlands Antilles	-5,286	-13,013	-28,881	-56,149	-106,733	-457	-772	-1,448	-2,614	-4,896	-397	-613	-1,088	-1,946	-3,588	-409	-881	-1,861	-3,337	-6,458
New Caledonia	8,256	21,631	19,797	25,430	20,175	195	562	458	542	746	135	400	322	380	528	287	823	680	800	1,100
New Zealand	244,292	289,892	210,526	137,154	157,617	4,984	5,954	6,533	6,899	7,487	3,612	4,339	4,769	5,033	5,520	7,040	8,381	9,172	9,692	10,477
Nicaragua	-31,878	-35,391	-77,794	-293,782	-472,363	-1,356	-1,456	-3,347	-13,036	-21,031	-436	-406	-1,136	-5,017	-8,174	-3,432	-3,903	-8,279	-30,441	-48,815
Niger	-25,548	-32,151	-98,481	-15,820	-55,650	-4,680	-5,975	-16,183	-2,120	-5,145	-2,700	-3,393	-10,601	624	-781	-7,486	-9,646	-23,928	-6,337	-11,776

	Net number of migrants					Net number of migrants with HbS														
						Median estimate					Lower-bound estimate					Higher-bound estimate				
**Country**	**1960**	**1970**	**1980**	**1990**	**2000**	**AS1960med**	**AS1970med**	**AS1980med**	**AS1990med**	**AS2000med**	**AS1960low**	**AS1970low**	**AS1980low**	**AS1990low**	**AS2000low**	**AS1960high**	**AS1970high**	**AS1980high**	**AS1990high**	**AS2000high**
Nigeria	-241,161	-217,821	894,658	10,340	84,609	-84,467	-83,924	87,590	-53,552	-70,692	-77,964	-78,428	64,733	-54,399	-73,777	-91,168	-89,396	115,123	-51,525	-65,559
Norway	-206,543	-144,033	-45,205	30,516	96,991	385	680	1,291	2,267	3,317	297	512	969	1,704	2,413	460	888	1,771	3,137	4,815
Oman	-9,534	-16,255	32,176	217,242	345,054	1,153	1,650	3,943	11,616	17,136	955	1,377	3,243	9,499	14,008	1,423	2,049	4,957	14,726	21,751
Pakistan	-2,494,514	-3,416,852	128,046	-190,837	-1,171,394	241,776	180,025	157,539	125,125	95,828	221,394	167,773	141,248	112,723	88,156	249,642	178,368	170,361	133,985	97,906
Panama	42,114	16,603	-62,532	-90,198	-91,903	717	-842	-5,141	-7,020	-8,273	450	-579	-3,401	-4,670	-5,517	1,368	-1,064	-7,614	-10,318	-12,126
Papua New Guinea	15,861	41,205	11,599	-1,065	-18,534	113	287	219	170	127	77	193	161	123	93	173	439	320	246	184
Paraguay	-132,193	-160,136	-147,530	-120,234	-221,534	-341	-54	1,565	2,256	1,024	65	374	1,741	2,291	1,510	-1,496	-1,404	369	1,230	-921
Peru	10,219	-3,090	-71,214	-236,209	-641,282	616	720	778	652	497	427	499	546	466	371	947	1,082	1,149	934	665
Philippines	63,655	-183,793	-860,688	-1,913,100	-2,772,024	241	966	657	1,076	3,898	190	768	490	836	2,904	318	1,244	882	1,385	5,274
Poland	-3,260,253	-3,123,949	-3,255,629	-3,065,719	-4,324,863	-1,045	-1,115	-840	-311	-429	449	323	443	683	863	-7,631	-7,300	-6,617	-5,219	-6,757
Portugal	-896,643	-1,182,656	-1,606,508	-1,345,179	-702,843	-14,414	-14,014	-6,385	8,547	34,372	-8,761	-7,757	-887	9,925	28,123	-25,204	-26,926	-20,534	159	37,621
Puerto Rico	-606,901	-783,566	-891,257	-1,135,437	-1,149,185	-3,406	-2,631	-2,051	-2,360	-1,055	-1,681	-719	-42	-314	902	-6,377	-6,025	-5,675	-5,820	-4,723
Qatar	11,856	66,246	143,416	350,988	455,455	203	808	2,741	6,195	7,977	155	538	1,983	4,289	5,584	289	1,294	4,010	9,580	12,192
Reunion	9,239	9,151	-43,266	55,487	-9,155	399	661	916	1,774	2,927	329	540	752	1,306	2,248	492	807	815	2,467	3,428
Romania	-256,977	-414,179	-432,041	-1,618,888	-1,175,209	410	-117	-415	-3,460	-1,882	539	310	106	-982	-218	-505	-1,798	-2,248	-10,853	-7,124
Russian Federation	-2,251,291	-3,247,172	-3,433,612	-2,671,655	1,673,738	922	820	920	1,322	4,209	221	169	189	294	2,187	3,500	3,336	3,767	5,296	10,391
Rwanda	-376,793	-419,261	-329,479	-284,989	121,690	-17,588	-16,477	-11,818	-9,208	75,364	-14,010	-13,047	-9,330	-7,201	67,128	-22,138	-20,843	-14,981	-11,792	83,964
Samoa	-7,207	-17,810	-49,723	-72,116	-94,481	3	12	14	6	13	1	9	10	5	9	3	16	19	12	18
Sao Tome and Principe	-15,898	-6,426	-8,368	-13,214	-28,298	-3,393	-1,741	-2,047	-2,685	-5,247	-1,064	-449	-569	-802	-1,774	-7,633	-4,124	-4,737	-6,127	-11,507
Saudi Arabia	11,960	275,554	1,663,224	4,499,346	4,826,856	-333	5,523	37,376	103,280	110,294	-496	3,603	26,311	73,643	78,412	50	8,954	56,414	153,921	164,827
Senegal	87,696	-19,724	-99,683	-101,613	-126,981	15,633	585	-9,191	-6,662	-9,899	11,896	-88	-8,121	-6,564	-9,200	20,042	1,397	-10,432	-6,737	-10,693
Serbia and Montenegro	-481,250	-838,636	-881,883	-1,149,880	-1,558,536	782	795	1,440	985	3,661	516	522	946	606	2,282	1,288	1,308	2,353	1,720	6,369
Sierra Leone	31,975	60,586	61,129	27,599	-60,519	7,280	14,170	13,737	4,572	-21,605	6,279	11,860	12,010	5,566	-12,966	8,255	16,504	15,389	3,274	-31,185
Singapore	491,003	426,307	376,127	569,036	1,064,355	3,160	2,336	2,006	2,220	5,480	2,671	1,983	1,692	1,851	4,553	3,808	2,801	2,419	2,757	6,795
Slovak Republic	-390,934	-360,540	-279,294	-329,545	-423,272	-384	-354	-270	-302	-253	-107	-96	-72	-66	-15	-1,335	-1,227	-945	-1,103	-1,096
Slovenia	-13,931	-22,180	-5,711	96,147	78,339	138	119	299	705	597	119	115	229	317	271	80	5	322	1,551	1,299
Solomon Islands	1,644	1,180	1,616	1,249	1,783	10	11	12	15	9	6	8	9	11	8	13	15	17	20	14
Somalia	-97,964	-108,026	-129,439	-173,858	-356,960	387	760	1,137	1,550	1,056	473	810	1,167	1,587	1,460	-26	349	685	945	-511
South Africa	738,717	727,503	732,478	827,748	310,966	28,657	26,858	24,775	35,795	39,058	21,866	20,416	18,744	27,493	31,002	38,564	36,402	33,900	48,072	49,800
Spain	-1,553,860	-2,083,518	-1,291,961	-753,830	638,129	-21,061	-27,936	-15,721	-6,801	16,643	-13,000	-17,189	-9,385	-3,606	12,666	-35,700	-47,473	-27,332	-12,687	23,139
Sri Lanka	935,628	957,639	264,753	-184,358	-385,522	44,547	48,004	27,877	18,349	15,190	38,193	41,165	23,920	15,784	13,090	52,674	56,748	32,965	21,671	17,917
St. Lucia	-8,478	-8,185	-13,732	-27,352	-39,436	-879	-876	-1,417	-2,769	-3,984	-579	-580	-940	-1,827	-2,619	-1,279	-1,269	-2,060	-4,049	-5,824
St. Vincent and the Grenadines	-14,544	-14,542	-24,418	-33,798	-49,496	-1,323	-1,314	-2,153	-3,009	-4,419	-653	-651	-1,075	-1,495	-2,189	-2,450	-2,434	-3,970	-5,554	-8,165
Sudan	120,795	71,938	56,732	-29,986	-143,090	16,120	11,827	12,249	10,406	3,751	13,915	10,422	11,029	10,112	5,147	18,576	13,237	13,299	9,948	929
Suriname	-2,340	-15,276	-64,220	-185,269	-216,647	-1,137	-1,542	-4,124	-11,225	-13,229	-454	-655	-1,859	-5,115	-5,994	-2,681	-3,497	-9,035	-24,464	-28,916
Swaziland	-26,415	-6,870	-19,360	-40,951	-13,132	-220	73	-117	176	653	-76	130	8	303	612	-490	-68	-360	-136	605
Sweden	-35,889	264,353	403,182	562,474	720,730	829	2,275	3,423	6,559	11,037	545	1,546	2,405	4,529	7,644	1,310	3,607	5,253	10,020	16,931
Switzerland	446,747	745,931	735,730	1,034,297	1,229,513	6,527	9,354	11,006	14,329	18,429	4,842	6,561	8,020	9,936	13,302	8,737	13,424	15,325	21,151	26,418
Syrian Arab Republic	-86,408	25,645	-265,298	-417,851	-510,261	-5,066	-6,466	-13,909	-21,732	-26,677	-4,088	-5,278	-10,848	-16,953	-20,820	-6,311	-7,918	-18,187	-28,435	-34,913
Tajikistan	204,651	251,787	219,009	100,719	-230,606	25	29	28	28	-8	8	10	11	10	1	72	90	73	41	-85
Tanzania	299,796	236,673	198,289	101,178	3,690	10,794	4,352	4,566	-3,118	-10,004	7,313	1,910	1,763	-4,000	-9,555	15,947	8,235	8,992	-1,314	-9,832
Thailand	306,560	179,979	135,608	-12,423	-22,455	256	217	652	1,144	3,760	197	164	472	823	2,688	336	302	934	1,629	5,377
Timor-Leste	6,285	7,242	4,259	5,368	-6,448	6	6	6	6	8	4	5	4	5	5	10	12	11	12	12
Togo	-106,280	-40,403	-52,167	-8,806	-76,344	-25,647	-12,291	-15,043	-5,729	-20,608	-22,013	-10,583	-12,948	-4,967	-17,715	-29,681	-14,203	-17,390	-6,602	-23,832
Tonga	-2,106	-2,666	-15,570	-25,475	-43,335	1	3	15	28	14	0	2	10	19	9	1	5	20	41	20
Trinidad and Tobago	-1,837	-2,165	-51,458	-159,375	-266,543	-1,097	-925	-4,616	-12,409	-20,252	-1,092	-1,035	-3,790	-9,021	-14,251	-608	-233	-5,060	-16,582	-28,295
Tunisia	-67,228	-297,451	-475,850	-529,858	-534,422	-1,733	-6,574	-10,377	-11,699	-11,792	-1,894	-5,595	-8,739	-9,833	-9,900	-1,122	-7,668	-12,285	-13,889	-14,007
Turkey	658,526	202,052	-1,611,500	-1,388,573	-1,742,086	16,724	9,749	-11,418	-9,034	-14,455	11,192	5,605	-10,541	-9,761	-14,112	27,075	17,693	-13,022	-7,220	-14,701
Turkmenistan	-24,436	144,745	149,132	-5,233	-104,096	-41	107	100	105	47	-10	69	64	88	56	-162	163	143	55	-70
Uganda	684,348	649,560	384,532	208,112	-133,237	70,318	65,125	32,497	10,650	-32,783	60,523	56,233	28,280	9,363	-25,890	82,059	75,850	37,873	12,709	-40,135
Ukraine	-2,236,059	-924,264	-252,145	-273,347	-710,620	-1,642	-1,554	-1,506	-1,635	116	-503	-470	-459	-467	693	-5,241	-4,962	-4,835	-5,383	-2,366
United Arab Emirates	-11,531	37,846	656,692	1,225,902	2,127,179	-46	1,488	18,102	33,640	57,954	-17	1,178	13,960	25,939	44,649	-100	1,963	24,588	45,726	78,842
United Kingdom	-1,800,451	-897,657	-729,887	-454,844	826,983	-22,004	-4,789	19,990	29,742	69,583	-14,763	-1,334	19,492	28,405	60,770	-31,354	-6,408	23,459	34,160	83,927
United States	9,794,528	10,585,151	14,521,558	21,059,969	32,065,983	83,684	128,672	232,617	384,075	637,312	51,578	80,065	153,199	259,205	444,580	143,688	216,119	369,559	594,670	955,415
Uruguay	113,917	48,576	-65,629	-125,222	-147,365	2,121	1,653	963	683	578	1,451	1,150	723	572	532	3,252	2,450	1,239	700	452
Uzbekistan	455,710	628,194	717,560	524,719	-301,483	40	432	546	643	456	17	238	303	366	281	94	841	1,050	1,180	705
Vanuatu	1,697	1,199	-385	-2,790	-5,242	28	31	29	15	6	20	23	21	11	3	40	46	41	25	8
Venezuela, RB	452,332	475,922	789,543	803,458	680,546	6,132	7,237	11,219	10,607	7,135	3,417	3,952	5,771	4,916	2,030	11,150	13,209	21,263	21,264	16,998
Vietnam	-267,812	-247,154	-549,903	-1,072,749	-1,708,808	17	17	17	-10	157	18	19	22	10	144	-2	2	-20	-91	91
Yemen, Rep.	74,503	-89,777	-36,214	-222,076	-261,379	2,647	1,905	2,925	2,292	2,667	1,764	1,328	1,995	1,645	1,907	4,185	2,919	4,549	3,414	3,981
Zambia	250,961	155,082	89,856	-33,411	-36,254	11,283	-150	-6,954	-18,895	-18,305	6,864	-2,445	-7,368	-17,397	-16,777	17,623	3,491	-5,883	-20,292	-19,749
Zimbabwe	225,675	318,516	247,997	137,402	36,359	19,225	25,902	22,316	17,148	12,142	15,077	20,282	17,512	13,546	9,689	25,252	34,031	29,288	22,445	15,816

Supplementary Table 3: Relative net number of migrants and estimated relative net number of migrants with HbS (median, low and high estimates) per country based on the World Bank Global Bilateral Migration Database and HbS estimates published by Piel et al (2013).

	Relative net number of migrants					Relative net number of migrants with HbS														
						Median estimate					Lower-bound estimate					Higher-bound estimate				
Country	1960	1970	1980	1990	2000	AS1960med	AS1970med	AS1980med	AS1990med	AS2000med	AS1960low	AS1970low	AS1980low	AS1990low	AS2000low	AS1960high	AS1970high	AS1980high	AS1990high	AS2000high
Afghanistan	-100	-208	-838	-2651	-2936	100	120	25	-17	0	84	102	24	5	21	120	140	11	-99	-87
Albania	-100	-20	38	-524	-3546	100	439	702	-1278	-12171	192	426	609	-647	-7634	-10	499	887	-2203	-19099
Algeria	-100	-358	-345	-343	-296	-100	-368	-355	-353	-305	-85	-279	-268	-266	-229	-109	-502	-486	-486	-420
Angola	100	-370	-821	-1075	-1478	-100	-212	-367	-475	-658	-82	-161	-274	-355	-492	-112	-270	-479	-621	-860
Argentina	100	81	65	49	39	100	76	57	44	31	63	48	36	27	19	168	130	98	75	55
Armenia	-100	-33	-21	-122	-339	-100	-26	-3	-156	2835	-26	-6	0	-44	2194	-429	-124	-50	-653	3050
Aruba	100	103	41	85	328	100	104	51	97	273	59	61	25	56	171	177	179	98	174	456
Australia	100	146	174	212	232	100	156	179	194	202	70	109	126	137	144	150	233	264	287	296
Austria	100	32	68	196	314	100	61	147	78	113	69	44	96	58	83	133	73	218	102	155
Azerbaijan	-100	-549	-1293	-2294	-4623	-100	-156	-248	-375	-634	-18	-33	-54	-85	-153	-416	-665	-990	-1499	-2486
Bahrain	100	138	355	616	783	100	81	323	677	361	88	84	279	575	415	131	91	435	891	403
Bangladesh	100	81	-698	-688	-661	100	109	84	96	109	86	94	87	97	108	118	128	62	76	93
Barbados	-100	-64	-247	-347	-328	-100	-57	-237	-344	-322	-30	-7	-70	-110	-98	-276	-190	-656	-923	-883
Belarus	-100	-131	-116	-102	-69	100	54	69	86	62	38	26	31	36	25	242	61	104	146	107
Belgium	-100	39	89	89	99	-100	40	92	100	121	-87	21	59	67	84	-111	80	153	163	187
Belize	100	-1125	-538	-2325	-983	-100	-680	-457	-1007	-1049	-72	-476	-322	-701	-753	-135	-959	-639	-1439	-1445
Benin	-100	-161	-520	-302	-351	-100	-157	-471	-291	-356	-85	-133	-399	-247	-302	-114	-180	-543	-333	-406
Bhutan	100	126	74	97	218	100	135	179	241	329	85	116	155	209	283	118	159	207	280	385
Bolivia	-100	-112	-139	-214	-349	100	133	157	193	284	73	97	117	145	212	138	182	213	259	377
Bosnia and Herzegovina	-100	-167	-140	-374	-606	-100	-170	-139	-350	-603	-30	-53	-40	-90	-178	-298	-500	-418	-1095	-1804
Botswana	-100	-78	-129	-79	23	-100	-48	-452	748	3126	46	63	-211	754	2518	-482	-337	-994	531	3829
Brazil	100	80	63	25	-19	100	68	42	-21	-122	61	39	20	-26	-103	171	124	86	-6	-140
Brunei Darussalam	100	140	218	-71	270	100	203	391	575	822	78	166	291	444	638	138	269	547	781	1122
Bulgaria	-100	-94	-80	-133	-143	-100	-94	-81	-132	-147	-43	-41	-35	-58	-61	-226	-212	-182	-298	-339
Burkina Faso	-100	-178	-185	-161	-206	-100	-179	-182	-136	-188	-89	-159	-163	-127	-180	-112	-201	-201	-144	-191
Burundi	-100	-205	-325	-235	-238	-100	-539	-1024	-675	-725	49	-295	-668	-420	-471	-344	-910	-1548	-1054	-1099
Cambodia	100	83	-25	-57	-13	100	81	-1	6	57	67	53	0	6	40	163	133	-3	2	84
Cameroon	100	115	16	88	38	100	115	16	62	16	88	101	13	52	12	115	132	20	75	22
Canada	100	130	158	204	258	100	149	200	271	362	74	110	149	197	268	143	211	282	390	512
Cape Verde	-100	-93	-163	-222	-416	-100	-88	-175	-229	-468	-66	-57	-119	-177	-351	-145	-130	-249	-288	-609
Central African Republic	100	-70	70	129	-28	100	-45	80	128	-14	99	-15	91	128	2	96	-89	59	123	-39
Chad	-100	-201	-381	-613	-388	100	72	13	-67	54	110	93	53	-2	95	82	40	-45	-158	-6
Chile	-100	-209	-464	-549	-478	100	75	69	92	130	65	49	46	63	89	161	122	109	140	200
China	-100	-56	-90	-96	-123	100	52	-44	57	652	111	65	-3	78	557	26	10	-157	-29	719
Colombia	-100	-201	-494	-725	-1116	-100	-207	-534	-789	-1238	-47	-102	-283	-424	-680	-198	-400	-987	-1450	-2240
Comoros	-100	-73	-11	-53	-84	-100	-61	56	4	-31	-59	-31	61	24	2	-178	-119	40	-41	-100
Congo, Dem. Rep.	100	98	37	4	-48	-100	-36	-89	-136	-287	-92	-39	-83	-123	-257	-105	-28	-92	-147	-318
Congo, Rep.	-100	-59	107	239	-132	-100	-80	65	157	-113	-78	-61	61	142	-88	-128	-105	66	171	-145
Costa Rica	100	-325	27	1283	3747	100	-73	81	673	1809	55	-31	40	285	760	184	-164	152	1483	4008
Cote d'Ivoire	100	165	210	216	258	100	164	208	222	260	86	140	178	190	226	116	191	242	256	296
Croatia	-100	-256	-200	130	-2	100	-214	143	343	108	186	87	326	185	99	-302	-1225	-658	660	17
Cuba	100	-267670	-408064	-487622	-580011	-100	-848	-1199	-1401	-1655	-40	-471	-681	-801	-949	-214	-1517	-2113	-2457	-2895
Cyprus	-100	-126	-178	-252	-176	-100	-148	-247	-386	-236	-13	-35	-85	-157	-59	-279	-376	-566	-834	-592
Czech Republic	-100	-98	-86	-16	-37	-100	-98	-86	-16	-11	-28	-28	-24	2	14	-360	-354	-312	-98	-132
Denmark	-100	-68	-69	-1	97	100	95	227	513	898	81	72	177	376	640	79	92	257	683	1287
Djibouti	100	195	375	646	812	100	180	367	578	742	80	146	294	463	595	130	237	476	754	965
Dominican Republic	100	84	-27	-240	-607	100	112	83	20	-99	81	98	93	74	32	116	112	28	-136	-422
Ecuador	-100	-134	-280	-539	-1865	100	191	292	318	357	68	128	199	224	281	143	277	416	421	308
Egypt, Arab Rep.	100	-1841	-7090	-14319	-17185	100	-53	-438	-954	-1177	102	17	-181	-438	-548	78	-206	-954	-1974	-2414
El Salvador	-100	-133	-424	-1770	-2952	100	135	-2074	-11120	-18794	-39	-34	-1380	-6994	-11810	443	525	-3063	-17493	-29602
Equatorial Guinea	100	-27	-123	-205	-366	-100	-643	-1061	-1631	-2908	-84	-511	-835	-1291	-2304	-110	-777	-1296	-1991	-3550
Eritrea	-100	-115	-122	-137	-152	-100	-86	-77	-35	-87	-24	-2	10	54	19	-286	-294	-298	-275	-361
Estonia	100	109	202	246	24	100	153	189	247	226	37	53	79	89	111	289	395	532	711	479
Ethiopia	100	90	86	67	38	100	94	97	98	97	78	74	76	77	77	135	127	132	132	129
Fiji	100	27	-124	-525	-963	100	69	42	35	41	82	56	35	29	34	125	86	52	44	51
Finland	-100	-140	-123	-105	-97	100	115	161	285	742	76	86	112	209	545	133	160	240	404	1076
France	100	175	194	195	196	100	291	504	560	306	74	208	377	430	223	154	443	716	772	455
French Guiana	100	226	314	754	1187	100	197	286	657	1022	65	133	190	437	681	158	296	438	1012	1571
French Polynesia	100	541	505	1495	1244	100	346	842	1052	1275	71	240	590	742	892	148	515	1246	1571	1869
Gabon	100	191	336	657	1036	100	583	1032	2251	3704	81	474	836	1838	3038	133	717	1279	2747	4492

	Relative net number of migrants					Relative net number of migrants with HbS														
						Median estimate					Lower-bound estimate					Higher-bound estimate				
Country	1960	1970	1980	1990	2000	AS1960med	AS1970med	AS1980med	AS1990med	AS2000med	AS1960low	AS1970low	AS1980low	AS1990low	AS2000low	AS1960high	AS1970high	AS1980high	AS1990high	AS2000high
Gambia, The	100	170	282	480	684	100	111	297	518	738	75	80	230	409	583	128	151	374	640	914
Georgia	100	-31	-88	-185	-334	100	-13	-77	-160	-304	38	4	-10	-38	-73	300	-152	-365	-702	-1227
Germany	100	225	340	346	453	-100	271	564	465	1060	-35	228	450	381	840	-186	383	807	659	1454
Ghana	100	68	-99	-83	-95	100	52	-69	-60	-71	86	44	-63	-56	-67	116	63	-74	-63	-74
Greece	-100	-120	-111	-71	-35	-100	-122	-119	-97	-78	-77	-93	-91	-75	-63	-138	-168	-164	-133	-103
Grenada	-100	-98	-199	-245	-311	-100	-97	-193	-240	-307	-43	-42	-86	-104	-131	-211	-205	-402	-500	-647
Guadeloupe	-100	-23	-188	75	-388	-100	-38	-202	28	-439	-72	-27	-146	19	-316	-139	-52	-281	42	-609
Guam	100	97	122	406	128	100	19	104	131	93	78	14	82	103	73	129	28	134	170	122
Guatemala	100	-133	-587	-2699	-5277	100	27	-47	-364	-747	58	22	-21	-196	-407	173	34	-98	-669	-1360
Guinea	-100	-124	-166	-181	-96	-100	-125	-168	-191	-145	-80	-100	-135	-155	-121	-120	-150	-202	-228	-168
Guinea-Bissau	-100	-119	-111	-182	-199	-100	-136	-111	-298	-341	-54	-79	-60	-198	-229	-175	-227	-195	-448	-505
Guyana	-100	-44	-537	-1240	-1781	-100	-45	-365	-847	-1219	-38	-13	-149	-356	-512	-245	-125	-859	-1967	-2833
Haiti	-100	-157	-227	-391	-591	-100	-152	-219	-375	-565	-76	-115	-165	-283	-426	-130	-200	-288	-495	-746
Honduras	100	-424	-496	-980	-2528	-100	-221	-248	-472	-1101	-52	-121	-137	-262	-606	-183	-392	-437	-829	-1953
Hong Kong SAR, China	100	103	115	113	123	100	178	129	205	875	70	135	91	149	691	152	243	195	294	1154
Hungary	-100	-104	-97	-99	-136	100	30	20	15	12	75	23	15	11	10	135	37	23	15	10
Iceland	-100	-81	-104	-114	-89	100	169	238	425	794	69	119	163	306	550	175	288	388	669	1194
India	100	263	384	-243	-999	-100	-87	-85	-94	-111	-90	-79	-75	-83	-97	-108	-93	-93	-105	-126
Indonesia	100	60	16	-40	-110	100	63	31	5	16	83	52	27	7	14	124	77	36	-1	12
Iran, Islamic Rep.	-100	-119	-166	69	-50	-100	-134	-295	-357	-496	-61	-83	-183	-224	-315	-164	-220	-478	-565	-773
Iraq	-100	-177	-340	-545	-927	-100	-157	-309	-490	-933	-37	-71	-151	-244	-479	-250	-359	-665	-1042	-1922
Ireland	-100	-213	-148	-158	-158	100	43	886	893	2582	257	408	963	986	2429	-700	-1688	-191	-271	1595
Israel	100	116	109	115	159	100	0	160	-712	-1520	-67	-165	-9	-622	-1212	689	660	802	-449	-1498
Italy	-100	-110	-84	-58	-25	-100	-101	-59	-7	53	-59	-58	-28	10	53	-178	-186	-121	-46	46
Jamaica	-100	-131	-214	-324	-480	-100	-131	-210	-313	-464	-79	-104	-166	-246	-365	-126	-166	-267	-399	-592
Japan	100	86	57	101	232	100	205	304	1028	3369	77	161	236	797	2629	131	269	400	1363	4422
Jordan	-100	-237	-402	-592	-608	-100	-240	-412	-619	-663	-69	-166	-286	-433	-465	-148	-354	-603	-895	-955
Kazakhstan	100	152	150	97	-65	100	134	137	134	79	33	45	46	46	28	310	421	429	424	250
Kenya	-100	-95	-67	-64	68	-100	-108	-67	-54	275	-91	-100	-64	-52	218	-110	-116	-69	-55	342
Korea, Dem. Rep.	-100	-111	-385	-424	-371	100	189	211	478	1200	78	133	133	356	922	156	244	256	600	1544
Korea, Rep.	-100	-87	-80	-144	-191	100	44	78	88	290	77	32	58	63	218	131	60	110	120	399
Kuwait	100	1563	3153	5192	4489	-100	657	1359	1823	1318	-15	532	1074	1516	1240	-282	781	1697	2106	1166
Kyrgyz Republic	100	100	97	29	-78	100	104	102	85	142	32	32	36	32	106	302	298	287	208	143
Lao PDR	-100	-237	-3655	-8734	-10281	100	100	67	-133	-167	100	100	100	67	33	200	167	-233	-900	-1200
Latvia	100	102	139	181	41	100	124	136	169	146	34	41	47	59	73	321	369	427	524	296
Lebanon	-100	-145	-339	-307	-292	-100	-142	-319	-408	-450	-69	-97	-219	-280	-309	-142	-201	-452	-574	-633
Lesotho	-100	-95	-107	-161	-86	-100	-99	-122	-170	-77	-20	-24	-32	-37	-9	-333	-319	-372	-547	-278
Liberia	100	216	-93	-98	-1468	100	220	274	272	-165	79	175	214	212	-148	122	267	331	329	-205
Libya	-100	567	3112	5042	5623	-100	3	358	624	659	-80	-30	187	348	352	-115	94	694	1147	1260
Lithuania	-100	-129	-25	17	-221	100	107	121	135	35	34	37	41	46	34	297	304	370	415	-20
Luxembourg	100	233	375	622	891	100	179	316	527	710	75	126	219	374	500	147	275	483	785	1059
Macao SAR, China	100	-3008	166	40	238	100	26	151	295	208	74	28	105	192	144	154	-13	226	456	323
Macedonia, FYR	-100	-133	-446	-128	-230	-100	-132	-436	-128	-228	-52	-68	-228	-52	-102	-193	-253	-834	-278	-473
Madagascar	100	48	-22	-37	-22	100	12	-252	-317	-232	65	-3	-227	-283	-208	162	41	-276	-350	-254
Malawi	100	-944	1293	440	2445	-100	-1347	126	-872	1558	2018	1120	1946	1225	2876	-4972	-6749	-3995	-5418	-1713
Malaysia	-100	254	159	215	207	100	1288	867	384	764	87	1095	733	306	613	114	1543	1047	502	994
Maldives	100	108	81	34	138	100	118	35	35	55	83	98	28	20	40	125	150	60	53	83
Mali	-100	-249	-523	-516	-654	-100	-418	-1003	-992	-1259	-77	-328	-790	-782	-990	-123	-521	-1253	-1239	-1581
Malta	-100	-118	-124	-131	-78	-100	-122	-126	-133	-82	-49	-58	-61	-64	-39	-205	-254	-260	-273	-172
Martinique	-100	-39	-441	63	-557	-100	-63	-427	-27	-596	-73	-46	-311	-21	-435	-137	-84	-581	-33	-810
Mauritania	-100	-74	-109	-102	-108	-100	-65	-91	-57	-57	-50	-21	-25	17	22	-186	-143	-211	-199	-212
Mauritius	-100	-427	-3211	-5261	-2680	100	101	125	87	379	79	82	110	87	328	121	114	82	-11	361
Mayotte	100	216	462	1117	3178	100	218	472	1132	2989	70	153	339	846	2107	144	321	698	1586	4467
Mexico	-100	-202	-556	-1058	-2177	-100	-236	-702	-1360	-2835	-86	-190	-551	-1066	-2209	-114	-294	-907	-1763	-3701
Micronesia, Fed. Sts.	100	59	-144	-94	-262	100	91	59	23	21	75	65	40	18	16	153	134	81	30	25
Moldova	-100	-37	55	-12	-188	-100	-98	-76	-133	-179	-13	-11	-3	-18	-31	-453	-459	-393	-615	-790
Mongolia	100	78	105	143	102	100	117	150	167	233	67	100	117	133	150	117	167	217	250	317
Morocco	-100	-250	-415	-562	-553	100	-72	-172	-247	-245	104	-7	-55	-86	-86	23	-265	-492	-680	-671
Mozambique	-100	-98	-70	-70	-61	-100	-98	-71	-77	-83	-77	-76	-55	-59	-64	-129	-126	-91	-98	-106
Myanmar	100	50	-38	-44	-148	100	95	63	41	31	85	81	54	35	28	119	112	74	47	35
Namibia	100	105	57	79	704	100	158	250	401	405	77	122	193	310	311	127	200	315	505	518
Nepal	-100	-140	-211	-93	-96	100	98	65	128	179	91	90	62	116	161	105	100	60	136	192

	Relative net number of migrants					Relative net number of migrants with HbS														
						Median estimate					Lower-bound estimate					Higher-bound estimate				
Country	1960	1970	1980	1990	2000	AS1960med	AS1970med	AS1980med	AS1990med	AS2000med	AS1960low	AS1970low	AS1980low	AS1990low	AS2000low	AS1960high	AS1970high	AS1980high	AS1990high	AS2000high
Netherlands	-100	-220	-119	229	408	-100	-100	-64	56	163	-72	-73	-52	21	100	-135	-131	-65	149	302
Netherlands Antilles	-100	-246	-546	-1062	-2019	-100	-169	-317	-572	-1071	-87	-134	-238	-426	-785	-89	-193	-407	-730	-1413
New Caledonia	100	262	240	308	244	100	288	235	278	383	69	205	165	195	271	147	422	349	410	564
New Zealand	100	119	86	56	65	100	119	131	138	150	72	87	96	101	111	141	168	184	194	210
Nicaragua	-100	-111	-244	-922	-1482	-100	-107	-247	-961	-1551	-32	-30	-84	-370	-603	-253	-288	-611	-2245	-3600
Niger	-100	-126	-385	-62	-218	-100	-128	-346	-45	-110	-58	-73	-227	13	-17	-160	-206	-511	-135	-252
Nigeria	-100	-90	371	4	35	-100	-99	104	-63	-84	-92	-93	77	-64	-87	-108	-106	136	-61	-78
Norway	-100	-70	-22	15	47	100	177	335	589	862	77	133	252	443	627	119	231	460	815	1251
Oman	-100	-170	337	2279	3619	100	143	342	1007	1486	83	119	281	824	1215	123	178	430	1277	1886
Pakistan	-100	-137	5	-8	-47	100	74	65	52	40	92	69	58	47	36	103	74	70	55	40
Panama	100	39	-148	-214	-218	100	-117	-717	-979	-1154	63	-81	-474	-651	-769	191	-148	-1062	-1439	-1691
Papua New Guinea	100	260	73	-7	-117	100	254	194	150	112	68	171	142	109	82	153	388	283	218	163
Paraguay	-100	-121	-112	-91	-168	-100	-16	459	662	300	19	110	511	672	443	-439	-412	108	361	-270
Peru	100	-30	-697	-2311	-6275	100	117	126	106	81	69	81	89	76	60	154	176	187	152	108
Philippines	100	-289	-1352	-3005	-4355	100	401	273	446	1617	79	319	203	347	1205	132	516	366	575	2188
Poland	-100	-96	-100	-94	-133	-100	-107	-80	-30	-41	43	31	42	65	83	-730	-699	-633	-499	-647
Portugal	-100	-132	-179	-150	-78	-100	-97	-44	59	238	-61	-54	-6	69	195	-175	-187	-142	1	261
Puerto Rico	-100	-129	-147	-187	-189	-100	-77	-60	-69	-31	-49	-21	-1	-9	26	-187	-177	-167	-171	-139
Qatar	100	559	1210	2960	3842	100	398	1350	3052	3930	76	265	977	2113	2751	142	637	1975	4719	6006
Reunion	100	99	-468	601	-99	100	166	230	445	734	82	135	188	327	563	123	202	204	618	859
Romania	-100	-161	-168	-630	-457	100	-29	-101	-844	-459	131	76	26	-240	-53	-123	-439	-548	-2647	-1738
Russian Federation	-100	-144	-153	-119	74	100	89	100	143	457	24	18	20	32	237	380	362	409	574	1127
Rwanda	-100	-111	-87	-76	32	-100	-94	-67	-52	428	-80	-74	-53	-41	382	-126	-119	-85	-67	477
Samoa	-100	-247	-690	-1001	-1311	100	400	467	200	433	33	300	333	167	300	100	533	633	400	600
Sao Tome and Principe	-100	-40	-53	-83	-178	-100	-51	-60	-79	-155	-31	-13	-17	-24	-52	-225	-122	-140	-181	-339
Saudi Arabia	100	2304	13907	37620	40358	-100	1659	11224	31015	33121	-149	1082	7901	22115	23547	15	2689	16941	46223	49498
Senegal	100	-22	-114	-116	-145	100	4	-59	-43	-63	76	-1	-52	-42	-59	128	9	-67	-43	-68
Serbia and Montenegro	-100	-174	-183	-239	-324	100	102	184	126	468	66	67	121	77	292	165	167	301	220	814
Sierra Leone	100	189	191	86	-189	100	195	189	63	-297	86	163	165	76	-178	113	227	211	45	-428
Singapore	100	87	77	116	217	100	74	63	70	173	85	63	54	59	144	121	89	77	87	215
Slovak Republic	-100	-92	-71	-84	-108	-100	-92	-70	-79	-66	-28	-25	-19	-17	-4	-348	-320	-246	-287	-285
Slovenia	-100	-159	-41	690	562	100	86	217	511	433	86	83	166	230	196	58	4	233	1124	941
Solomon Islands	100	72	98	76	108	100	110	120	150	90	60	80	90	110	80	130	150	170	200	140
Somalia	-100	-110	-132	-177	-364	100	196	294	401	273	122	209	302	410	377	-7	90	177	244	-132
South Africa	100	98	99	112	42	100	94	86	125	136	76	71	65	96	108	135	127	118	168	174
Spain	-100	-134	-83	-49	41	-100	-133	-75	-32	79	-62	-82	-45	-17	60	-170	-225	-130	-60	110
Sri Lanka	100	102	28	-20	-41	100	108	63	41	34	86	92	54	35	29	118	127	74	49	40
St. Lucia	-100	-97	-162	-323	-465	-100	-100	-161	-315	-453	-66	-66	-107	-208	-298	-146	-144	-234	-461	-663
St. Vincent and the Grenadines	-100	-100	-168	-232	-340	-100	-99	-163	-227	-334	-49	-49	-81	-113	-165	-185	-184	-300	-420	-617
Sudan	100	60	47	-25	-118	100	73	76	65	23	86	65	68	63	32	115	82	83	62	6
Suriname	-100	-653	-2744	-7917	-9258	-100	-136	-363	-987	-1164	-40	-58	-164	-450	-527	-236	-308	-795	-2152	-2543
Swaziland	-100	-26	-73	-155	-50	-100	33	-53	80	297	-35	59	4	138	278	-223	-31	-164	-62	275
Sweden	-100	737	1123	1567	2008	100	274	413	791	1331	66	186	290	546	922	158	435	634	1209	2042
Switzerland	100	167	165	232	275	100	143	169	220	282	74	101	123	152	204	134	206	235	324	405
Syrian Arab Republic	-100	30	-307	-484	-591	-100	-128	-275	-429	-527	-81	-104	-214	-335	-411	-125	-156	-359	-561	-689
Tajikistan	100	123	107	49	-113	100	116	112	112	-32	32	40	44	40	4	288	360	292	164	-340
Tanzania	100	79	66	34	1	100	40	42	-29	-93	68	18	16	-37	-89	148	76	83	-12	-91
Thailand	100	59	44	-4	-7	100	85	255	447	1469	77	64	184	321	1050	131	118	365	636	2100
Timor-Leste	100	115	68	85	-103	100	100	100	100	133	67	83	67	83	83	167	200	183	200	200
Togo	-100	-38	-49	-8	-72	-100	-48	-59	-22	-80	-86	-41	-50	-19	-69	-116	-55	-68	-26	-93
Tonga	-100	-127	-739	-1210	-2058	100	300	1500	2800	1400	0	200	1000	1900	900	100	500	2000	4100	2000
Trinidad and Tobago	-100	-118	-2801	-8676	-14510	-100	-84	-421	-1131	-1846	-100	-94	-345	-822	-1299	-55	-21	-461	-1512	-2579
Tunisia	-100	-442	-708	-788	-795	-100	-379	-599	-675	-680	-109	-323	-504	-567	-571	-65	-442	-709	-801	-808
Turkey	100	31	-245	-211	-265	100	58	-68	-54	-86	67	34	-63	-58	-84	162	106	-78	-43	-88
Turkmenistan	-100	592	610	-21	-426	-100	261	244	256	115	-24	168	156	215	137	-395	398	349	134	-171
Uganda	100	95	56	30	-19	100	93	46	15	-47	86	80	40	13	-37	117	108	54	18	-57
Ukraine	-100	-41	-11	-12	-32	-100	-95	-92	-100	7	-31	-29	-28	-28	42	-319	-302	-294	-328	-144
United Arab Emirates	-100	328	5695	10631	18447	-100	3235	39352	73130	125987	-37	2561	30348	56389	97063	-217	4267	53452	99404	171396
United Kingdom	-100	-50	-41	-25	46	-100	-22	91	135	316	-67	-6	89	129	276	-142	-29	107	155	381
United States	100	108	148	215	327	100	154	278	459	762	62	96	183	310	531	172	258	442	711	1142
Uruguay	100	43	-58	-110	-129	100	78	45	32	27	68	54	34	27	25	153	116	58	33	21
Uzbekistan	100	138	157	115	-66	100	1080	1365	1608	1140	43	595	758	915	703	235	2103	2625	2950	1763

Country	Relative net number of migrants					Relative net number of migrants with HbS: Median estimate					Lower-bound estimate					Higher-bound estimate				
	1960	1970	1980	1990	2000	AS1960med	AS1970med	AS1980med	AS1990med	AS2000med	AS1960low	AS1970low	AS1980low	AS1990low	AS2000low	AS1960high	AS1970high	AS1980high	AS1990high	AS2000high
Vanuatu	100	71	-23	-164	-309	100	111	104	54	21	71	82	75	39	11	143	164	146	89	29
Venezuela, RB	100	105	175	178	150	100	118	183	173	116	56	64	94	80	33	182	215	347	347	277
Vietnam	-100	-92	-205	-401	-638	100	100	100	-59	924	106	112	129	59	847	-12	12	-118	-535	535
Yemen, Rep.	100	-121	-49	-298	-351	100	72	111	87	101	67	50	75	62	72	158	110	172	129	150
Zambia	100	62	36	-13	-14	100	-1	-62	-167	-162	61	-22	-65	-154	-149	156	31	-52	-180	-175
Zimbabwe	100	141	110	61	16	100	135	116	89	63	78	105	91	70	50	131	177	152	117	82
